# AARS1-catalyzed H4K12 lactylation promotes HCC resistance to targeted therapy by activating RAPGEF3-RAP1 signaling

**DOI:** 10.1038/s41419-026-09011-6

**Published:** 2026-07-21

**Authors:** Tanlun Zeng, Wanwan Zhu, Guanqun Sun, Tengjiao Wang, Chenna Liu, Guanghui Hu, Junyu Liu, Pengyun Ling, Li Wang, Chang Fan, Terence Kin Wah Lee, Jianru Xiao, Xijun Liang, Jin Ding

**Affiliations:** 1 Clinical Cancer Institute, Center for Translational Medicine, Naval Medical University, Shanghai, China; 2 National Key Laboratory of Immunity and Inflammation, Institute of Immunology, Naval Medical University, Shanghai, China; 3 Center for Translational Medicine, Naval Medical University, Shanghai, China; 4 Department of Plastic and Reconstructive Surgery, Shanghai Fourth People’s Hospital, School of Medicine, Tongji University, Shanghai, China; 5 Department of Gastroenterology, The Third Affiliated Hospital of Sun Yat-Sen University, Guangzhou, China; 6 Department of Blood Transfusion, Naval Specialty Medical Center, Naval Medical University, Shanghai, China; 7 Department of Applied Biology and Chemical Technology, The Hong Kong Polytechnic University, Hong Kong, China; 8 Institute of Orthopedics, Department of Orthopedic Oncology, Shanghai Changzheng Hospital, Naval Medical University, Shanghai, China

**Keywords:** Liver cancer, Cancer therapeutic resistance

## Abstract

Although receptor tyrosine kinase inhibitors (sorafenib and lenvatinib) have been applied as a first-line targeted therapy for advanced unresectable hepatocellular carcinoma (HCC) for decades, their clinical efficacy is limited and the underlying mechanism remains unclear. HCC is a highly glycolytic malignancy characterized by excessive lactate accumulation in the tumor microenvironment (TME). Emerging evidences show that histone lactylation plays a critical role in various biological processes, but its function in receptor tyrosine kinase inhibitor resistance remains obscure. This study was designed to elucidate the role of histone lactylation in receptor tyrosine kinase inhibitor resistance in HCC. Clinical cohort analyses revealed that the increased nuclear pan-lysine lactylation (pan-Kla) predicts poor patient prognosis and high H4K12la level correlates with targeted drug resistance. Furthermore, lactate bidirectionally controls H4K12la through the opposing enzymatic activities of AARS1 (writer) and HDAC11 (eraser). Integrative CUT&Tag and ATAC-seq analyses demonstrated that H4K12la directly activates the promoter of RAPGEF3, a predominant upstream regulator of the RAP1 signaling pathway. Inhibition of RAPGEF3 reversed the lactate-induced targeted drug resistance both in vitro and in vivo, suggesting H4K12 lactylation modulates targeted drug resistance by activating RAPGEF3-RAP1 signaling. Notably, combining the RAPGEF3 inhibitor ESI-09 with lenvatinib synergistically suppressed HCC growth in mouse models. Clinico-pathological analyses revealed that elevated expression of the AARS1/H4K12la/RAPGEF3 axis correlated with inferior survival and sorafenib resistance in HCC patients, which was further confirmed in patient-derived xenograft (PDX) models. This study delineates a novel metabolic-epigenetic mechanism whereby lactate modulates hepatocellular carcinoma sensitivity to targeted therapies through histone lactylation and suggests AARS1-H4K12la-RAPGEF3 axis may serve as an interventional target to overcome targeted drug resistance, offering a promising strategy to enhance clinical outcomes in HCC patients.

**Figure:**
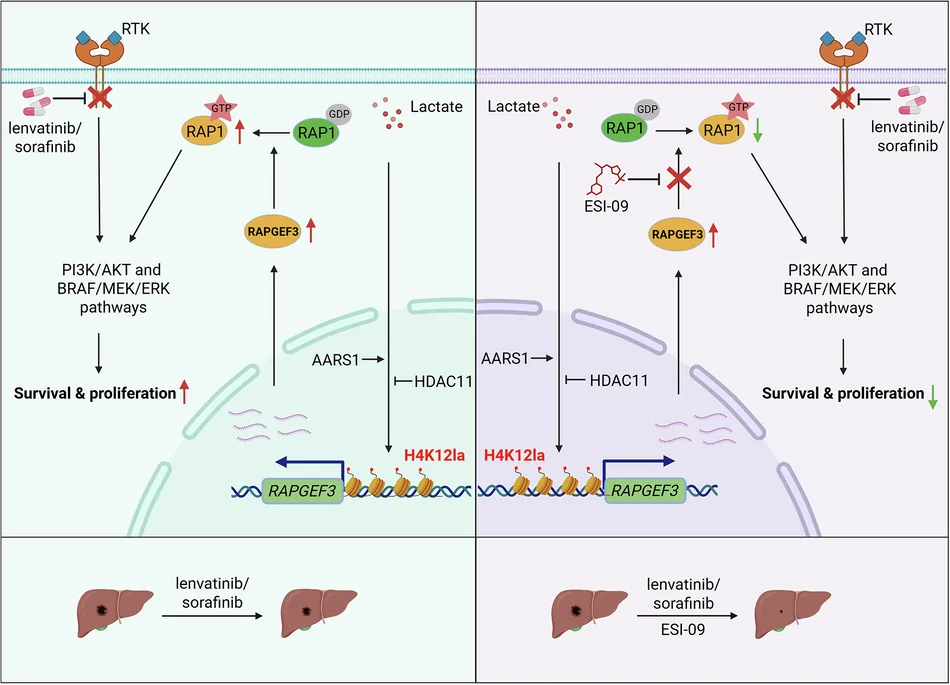

## Background

Hepatocellular carcinoma (HCC) represents the third most prevalent cause of cancer-associated mortality globally, with characteristically dismal clinical outcomes [1, 2]. The profoundly immunosuppressive nature of the HCC tumor microenvironment (TME) contributes substantially to the limited efficacy of immunotherapeutic interventions [3]. Consequently, molecular targeted agents - particularly the multi-kinase inhibitors sorafenib and lenvatinib - remain cornerstone therapies in the systemic management of advanced HCC [4]. Nevertheless, only a small proportion of patients respond to these targeted agents, and the mechanism underlying targeted drug resistance remains unclear. Systematic elucidation of the molecular pathways mediating targeted drug resistance is consequently imperative to enable precision medicine approaches for HCC management.

Metabolic reprogramming represents a fundamental hallmark of malignancy, functioning as a pivotal adaptive mechanism that enables neoplastic cells to sustain microenvironmental homeostasis [5]. In HCC, this metabolic rewiring is characterized by a pronounced shift towards anaerobic glycolysis (the Warburg effect) [6]. Such metabolic alterations not only facilitate rapid ATP generation to meet bioenergetic demands but also provide crucial biosynthetic precursors to support uncontrolled cell proliferation. Notably, the emerging evidences indicate that this metabolic shift might influence therapeutic vulnerability in cancer cells [7]. The glycolytic end-product lactate accumulates markedly within the hypoxic TME [8], functioning as both: (i) a pleiotropic oncometabolite that acidifies the TME and orchestrates malignant progression through bioenergetic and signaling network dysregulation; and (ii) a mediator of therapeutic resistance. It was reported that estrogen-related receptor alpha (ERRα)-regulated lactate metabolism contributes to resistance to mTOR inhibitor in breast cancer and MCT1-dependent lactate recycling determines colorectal cancer cells vulnerability upon EGFR targeted therapy [9, 10]. Lactate attenuates cellular responsiveness to EGFR inhibitor through pathological activation of the HGF/MET signaling cascade in non-small cell lung cancer (NSCLC) [11]. In gastric cancer, lactate-induced BDNF/TrkB signaling activation has been shown to compromise anlotinib efficacy [9]. The aforementioned literature supports that lactate may induce resistance to various platforms of anti-cancer therapy.

Lactylation, a recently characterized lactate-mediated post-translational modification, has emerged as a critical regulator of oncogenic processes. Non-histone protein lactylation (e.g., NBS1 and MRE11 lactylation) has been mechanistically linked to the development of tumor radioresistance and chemoresistance [10, 12]. Lactylation of H3K18 was reported to promote NNMT transcription and mediate EGFR-TKI resistance in NSCLC [13]. These findings collectively establish lactylation as a central mechanistic determinant of therapeutic response in cancer, while the effect of histone lactylation in targeted drug resistance of HCC has not been reported so far.

The landmark study by Zhao et al. first reported p300 as a lactyltransferase, albeit with limited catalytic efficiency due to the extremely low concentration of lactoyl-CoA compared to other acyl-CoA substrates [14, 15]. Our group, for the first time, demonstrated that AARS1 can directly utilize lactate as a substrate to catalyze lactylation with superior efficiency [16, 17]. While the identification of lactyltransferases remains an active area of investigation, the corresponding delactylases await comprehensive characterization. Although lactylation profiles have been delineated in HCC, a systematic elucidation of its regulatory circuitry - particularly its mechanistic contributions to receptor tyrosine kinase inhibitor resistance - represents a critical unmet need in the field.

Herein, we found that elevated nuclear lactylation in HCC cells and histone H4K12la promotes receptor tyrosine kinase inhibitor resistance. Mechanistically, AARS1-catalyzed H4K12la enhances RAPGEF3 transcription and then activates RAP1 kinase, thereby enabling the activation of downstream pathways of PI3K/AKT and BRAF/MEK/ERK, which compromises the inhibition of tyrosine kinases by targeted drugs. These results define AARS1/H4K12la/RAPGEF3 axis as a promising biomarker and therapeutic target for precision medicine in HCC.

## Materials and methods

### Patients and specimens

Cohort 1 consisted of 181 patients with HCC who underwent surgical resection at Naval Medical University Eastern Hepatobiliary Surgery Hospital (EHBH) between 2001 and 2009, with their clinicopathological characteristics summarized in Suppl.Table S1. To identify factors associated with sorafenib-related benefit in patients with recurrent HCC, 101 patients with post-surgical HCC recurrence from Naval Medical University EHBH were enrolled as Cohort 2 (clinicopathological data provided in Suppl. Table S2). Sorafenib was administered at a dose of 400 mg twice daily. Cohort 3 comprised 66 patients diagnosed with HCC who underwent surgical resection at Naval Medical University EHBH. Cohort 3 patients had 5-year follow-up information and were mainly used for prognosis and clinicopathological correlation analyses. Their clinicopathological characteristics were detailed in Suppl. Table S3.

### Animal studies

Male BALB/c nude mice (5–6 weeks old) were obtained from Jiangsu Jicui Yaokang Biotechnology Co., Ltd. and maintained in individually ventilated cages (IVC) at the Translational Medicine Research Center’s Clinical Cancer Institute (Naval Medical University) under standardized conditions. For tumor xenograft establishment, HCCLM3 cells in logarithmic growth phase (6 × 10⁶ cells in 100 μL suspension) were inoculated subcutaneously into the mice. When tumor volumes reached 100–200 mm³, animals were randomized into treatment groups receiving either: (1) intraperitoneal ESI-09 (20 mg/kg), (2) oral lenvatinib (20 mg/kg), (3) combination therapy, or (4) vehicle control, administered every other day. Tumor dimensions were measured every 48 h using digital calipers, and tumor volume was calculated using the formula (length × width²)/2. At study termination, tumors were excised, photographed, and weighed for comparative analysis. Randomization of mice into treatment and control groups was performed via a computer-generated schedule. Every two days, an investigator blinded to group allocation assessed tumor volumes. No animals were excluded from the analysis, nor were any unexpected adverse events observed. Sample sizes were informed by prior studies and feasibility, rather than a formal power analysis.

### Cell lines and culture

The human HCC cell lines HCCLM3 and HepG2 were obtained from the National Resource Center for Mode and Characteristic Experimental Cells and maintained under standard culture conditions (37 °C, 5% CO₂). The cell lines were routinely tested and confirmed to be free of mycoplasma, with authentication performed using short tandem repeat profiling based on ECACC or ATCC reference standards. Stable AARS1-knockout and AARS1-overexpressing HCC cell lines were generated via lentiviral transduction.

### 4D Label-free quantitative lactylation proteome assay

(1) Protein Extraction: Add an appropriate amount of protein lysis buffer to the protein samples and perform ultrasonic lysis. Centrifuge at 12,000 × g for 10 min, transfer the supernatant to a new centrifuge tube, and measure the protein concentration. (2) Trypsin Digestion: Take an equal amount of protein samples for enzymatic digestion and equalize the volumes between samples. Add TCA to the samples (final concentration 20%), mix well by shaking, and precipitate at 4 °C for 2 h. Centrifuge at 4500 × g for 5 min, discard the supernatant, and wash the precipitate with pre-cooled acetone 2–3 times, then air dry. Add TEAB to the precipitate (final concentration 200 mM), disperse with ultrasonication, and add trypsin (enzyme: protein ratio of 1:50) for overnight digestion. Add an appropriate amount of dithiothreitol (DTT, final concentration 5 mM) to the digested products, reduce at 56 °C for 30 min, and then add an appropriate amount of iodoacetamide (final concentration 11 mM), incubate in the dark at room temperature for 15 min. (3) Modification Enrichment: Dissolve the peptides in IP buffer, transfer the supernatant to lactylation resin (pre-washed), and place it on a shaker at 4 °C with gentle shaking overnight. Wash the resin with IP buffer 4 times, further wash with deionized water 2 times, and finally elute the resin-bound peptides with 0.1% trifluoroacetic acid (TFA) eluent 3 times, collect the eluate, and vacuum freeze-dry. After desalting according to the C18 ZipTips manual, vacuum freeze-dry again for liquid chromatography-mass spectrometry (LC-MS) analysis. (4) Liquid Chromatography-Mass Spectrometry (LC-MS) Analysis: Dissolve the peptides in mobile phase A and separate them in a NanoElute ultra-high-performance liquid chromatography (UHPLC) system. Mobile phase A: an aqueous solution containing 0.1% formic acid and 2% acetonitrile; Mobile phase B: a solution containing 0.1% formic acid and 100% acetonitrile. Set the UHPLC gradient (0–44 min, 6–22% B; 44–54 min, 22–30% B; 54–57 min, 30–80% B; 57–60 min, 80% B), with a flow rate maintained at 450 nL/minute. After separating the peptides with the UHPLC system, inject them into the Capillary ion source for ionization and analyze with a timsTOF Pro mass spectrometer (ion source voltage set to 1.7 kV). Set the secondary mass spectrometry scan range to 400–1500 m/z. Use the Parallel Accumulation Serial Fragmentation (PASEF) mode for data acquisition, after one first-level mass spectrum acquisition, perform 10 PASEF acquisitions for parent ions with charges ranging from 0 to 5, and set the dynamic exclusion time for tandem mass spectrometry scanning (30 s) to avoid repeated scanning of parent ions.

### Assay for transposase-accessible chromatin with high throughput sequencing (ATAC-seq)

(1) Cell Thawing and Counting: Retrieve cells stored in cryopreservation solution from −80 °C, thaw and melt them, and use a hemocytometer to count viable and non-viable cells separately. (2) Lysis, Transposition Reaction, and Purification: After counting, take the appropriate number of cells, resuspend and lyse them with lysis buffer, add transposase for the transposition reaction, and perform purification after the transposition is complete. (3) PCR Amplification: The transposition products are amplified by PCR using specific primers and then purified. (4) Single-Strand Separation and Circularization: The PCR products are heat-denatured into single strands, and then a bridging primer is used to circularize the single-stranded DNA into a single-stranded circular DNA library. (5) Sequencing: Perform sequencing using the obtained amplified product DNA nanoballs.

### Cleavage Under Targets and Tagmentation (CUT&Tag)

(1) Collected HCC cells are adsorbed and combined with Concanavalin A-coated magnetic beads. (2) Perforate HCC cells using the non-ionic detergent digitonin. (3) Successively incubate with the primary antibody against H4K12la (PTM BIO, Cat#PTM-1411RM), the corresponding secondary antibody, and the pA-Tn5 fusion protein (where pA in pA-Tn5 can recognize the Fc region of the secondary antibody, and Tn5 enzyme cuts the DNA sequence near the target protein upon the addition of Mg^2+^, additionally, Tn5 enzyme adds adapter sequences to both ends of the cut fragment). (4) The cutting products are subjected to PCR amplification for library construction and further purification. (5) High-throughput sequencing: After the library is qualified, different libraries are pooled according to the required effective concentration and target data output for Illumina NovaSeq sequencing. In the sequencing flow cell, four fluorescently labeled dNTPs, DNA polymerase, and adapter primers are added for amplification. The sequencing instrument captures the fluorescent signals, and computer software translates the light signals into sequencing peaks, ultimately obtaining the sequence information of the target fragments.

### RNA interference

Small interfering RNAs (siRNAs) were chemically synthesized by GenePharma (Shanghai, China), with target sequences detailed in Suppl. Table S4. HCC cells (HCCLM3 and HepG2) were transfected with these siRNAs using JetPEI DNA transfection reagent according to the manufacturer’s recommended protocol. Cells were harvested for subsequent analyses 24–72 h post-transfection.

### Real-time quantitative PCR

RNA was extracted using the RNAfast200 Kit (Fastagen Biotech, Cat#220011), and cDNA was synthesized by reverse transcription PCR. Target genes were amplified with qPCR SYBR Green Master Mix. Primer specificity was verified by melting curve analysis. Each sample was analyzed in triplicate under consistent conditions. The Ct values of target genes were normalized to β-actin, and relative expression levels were calculated using the 2^−^^ΔΔCt^ method. Primer sequences are provided in Suppl. Table S5.

### Western blot

Proteins, either histones or total proteins, were extracted using the method described previously [17], and the protein concentration was determined using the Pierce™ BCA Protein Assay Kit (Thermo Fisher Scientific, Cat#23227). After denaturation, the proteins were subjected to electrophoresis, transferred to a membrane, and blocked. The primary antibodies targeting L-lactyl lysine (PTM BIO, Cat#PTM-1401RM), H4K12la (PTM BIO, Cat#PTM-1411RM), AARS1 (Proteintech, Cat#17394-1-AP), RAPGEF3 (AiFang biological, Cat#AF301627), Histone H4 (Proteintech, Cat#16047-1-AP), GAPDH (CST, Cat#2118S), β-actin (CST, Cat#3700S), α-tubulin (Proteintech, Cat#66031-1-Ig), or HDAC11 (Proteintech, Cat#67949-1-Ig) were incubated overnight, followed by incubation with the secondary antibodies (CST, Cat#7074S; 7076S) at room temperature for 2 h. Finally, an SuperSignal™ West Femto Maximum Sensitivity Substrate (Thermo Fisher Scientific, Cat#34096) was used for detection. Uncropped Western blot images are available in the Supplementary material (titled Supplementary Material – Uncropped Gels for Western Blots).

### Flow cytometry

Cellular viability was assessed using a commercial apoptosis and necrosis detection kit (Biolegend, Cat#640930) with flow cytometric analysis. Briefly, harvested cell pellets were washed twice with ice-cold staining buffer and resuspended in 500 μL of fresh buffer. Cell suspensions were then stained with 2.5 μL Hoechst and 2.5 μL propidium iodide (PI), followed by thorough mixing. After 30 min of dark incubation on ice, samples were immediately analyzed by flow cytometry to assess the death of the HCC cells.

### RAP1 activity assay

RAP1 activity assays were conducted following the manufacturer’s instructions (Cell Biolabs, Cat#STA-406-1). In brief, migrating cells were lysed with the buffer provided in the kit. The cell lysates were mixed with 40 μL of Ral-GDS agarose beads and incubated for 4 h at 4 °C to capture active RAP1. The Ral-GDS beads were subsequently washed and boiled in 2× SDS sample buffer. Finally, the samples were analyzed by immunoblotting with the anti-RAP1 antibody provided in the kit.

### Immunohistochemical staining

Immunohistochemical staining of serial tissue sections was performed as previously described [17, 18]. Specifically, HCC tissues underwent fixation, dehydration, clarification, and paraffin impregnation before embedding and sectioning. Sections were heated at 60 °C for 1 h, deparaffinized, and subjected to endogenous peroxidase inactivation and antigen retrieval. After blocking, sections were incubated overnight with the primary antibodies targeting L-lactyl lysine (PTM BIO, Cat#PTM-1401RM), H4K12la (PTM BIO, Cat#PTM-1411RM), AARS1 (Santa Cruz, Cat#sc-165990), RAPGEF3 (Proteintech, Cat#12572-1-AP), or HDAC11 (Proteintech, Cat#67949-1-Ig), followed by a 2 h incubation with the secondary antibody (Boster Biological Technology, Catalog#SV0004) at room temperature. DAB (DAKO, Catalog#K346811) was used for coloration, followed by hematoxylin counterstaining and acid ethanol differentiation. Finally, sections were dehydrated scanned. The mean positive staining intensity was analyzed using Aperio Image Scope software. Nuclear staining was quantified by two senior pathologists in a blinded manner, with both staining intensity and positive area proportion scored on a 0.5–3 scale (0.5, 1, 1.5, 2, 2.5, 3) according to staining depth and positive proportion. The nuclear IHC score was derived from the product of the intensity score and positive range score.

### Statistical analysis

The data were analyzed with IBM SPSS Statistics 26.0 software, and the graphs were plotted with GraphPad Prism 9.5.0 software. Continuous variables that conformed to a normal distribution are expressed as the mean ± standard deviation, whereas those that did not conform to a normal distribution are expressed as the median (interquartile range, 25–75%). For two sets of quantitative data that conformed to a normal distribution, Student’s t test was used; otherwise, the Mann‒Whitney U test was applied. Categorical variables were compared with the chi-square test. For comparisons involving more than two groups, one-way ANOVA was performed. In some experiments, two-way ANOVA was performed. In the in vivo studies, n represents the number of individual mice used in the experiments. In the in vitro cell culture studies, n (represented as dots in bar charts) refers to the number of independent experiments conducted. Overall survival (OS) was defined as the time from the start of surgery until patient death; disease-free survival (DFS) was defined as the time from the start of surgery until disease recurrence or patient death. Survival curves were plotted with the Kaplan‒Meier method, and the log-rank test was used to compare survival rates (OS and DFS). The linear correlation between two variables was analyzed by Pearson or Spearman correlation analysis. All tests were two-tailed, and a significance level of *α* = 0.05 was used to define statistically significant differences.

## Results

### Increased nuclear pan-lysine lactylation (pan-Kla) predicts poor prognosis and therapy resistance in HCC

**Fig. 1:**
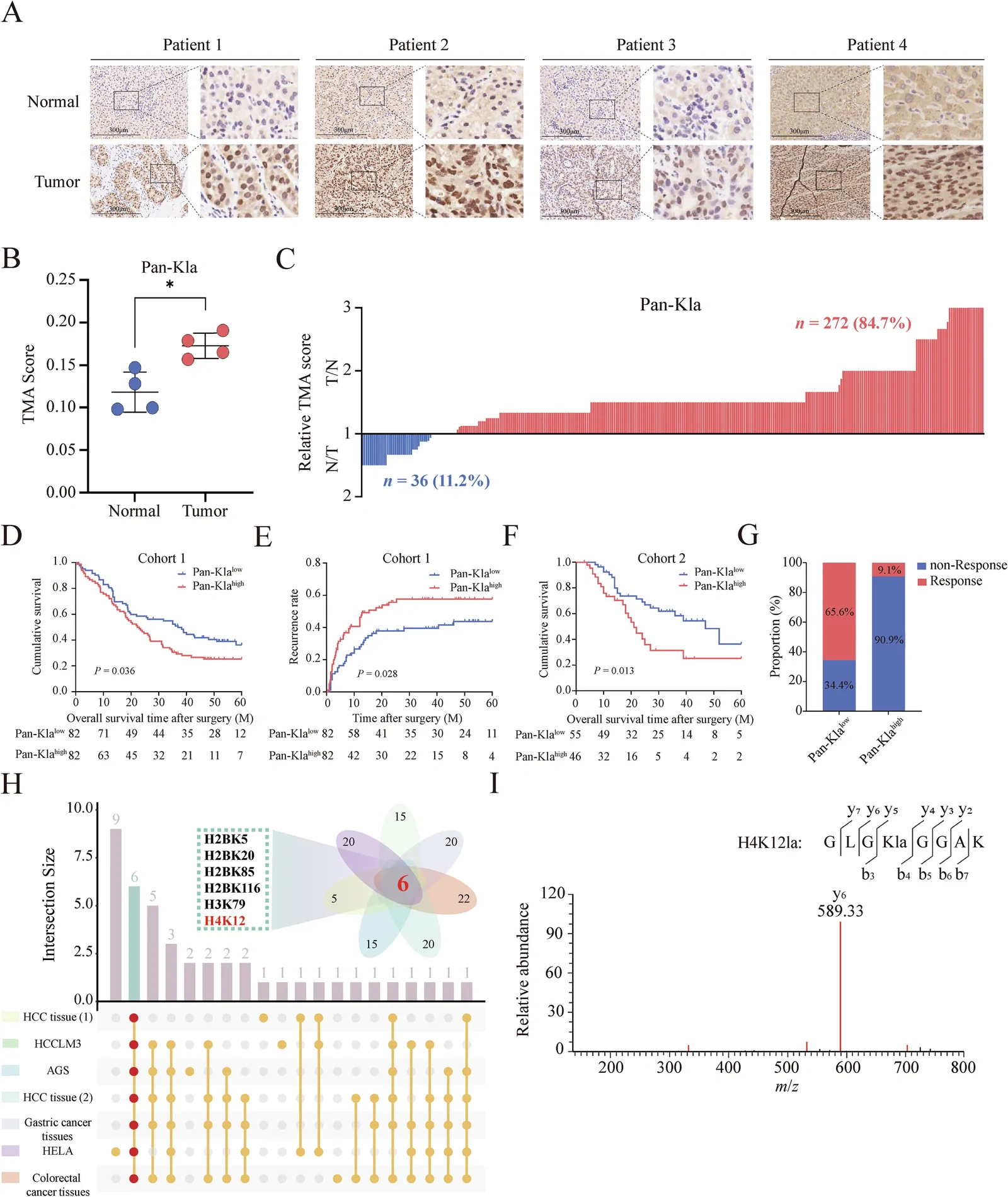
Increased levels of pan-Kla in the nuclei of HCC cells are associated with poor prognosis and resistance to receptor tyrosine kinase inhibitor. **A**, **B** IHC assessment of pan-Kla in HCC and normal liver tissues (Patient 1-4, from PLC cohort 1), with localization (**A**) and quantified scoring (**B**). Scale bar, 300 μm. **C** Nuclear pan-Kla levels in HCC and normal liver tissues from PLC cohorts 1–3, which comprised PLC patients from Naval Medical University EHBH. A total of 84.7% of patients had higher levels in HCC tissues (T/N > 1), and 11.2% of patients had higher levels in normal tissues (N/T > 1). **D** Kaplan‒Meier survival curves based on nuclear pan-Kla levels in HCC patients (PLC cohort 1) analyzed by the log-rank test (OS). **E** Kaplan‒Meier curves for recurrence risk based on nuclear pan-Kla levels in HCC patients (PLC cohort 1) analyzed by the log-rank test (DFS). **F** Kaplan‒Meier survival curves based on nuclear pan-Kla levels in sorafenib-treated HCC patients (PLC cohort 2) analyzed by the log-rank test (OS). **G** Correlation between nuclear pan-Kla levels in HCC (PLC cohort 2) and sorafenib efficacy (χ^2^ = 10.471, *P* = 0.001). Patients with disease progression (PD) were considered to demonstrate ineffective treatment; those with complete response (CR), partial response (PR), and stable disease (SD) were considered to demonstrate effective treatment. **H** Upset and Venn diagrams of histone lactylation sites in HCCLM3 cells, HeLa cells, AGS cells, HCC tissues, gastric cancer tissues, and colorectal cancer tissues. Six sites (H2BK5, H2BK20, H2BK85, H2BK116, H3K79, and H4K12) were common across all cells and tissues. **I** LC‒MS/MS detection of peptide modifications in HCCLM3 cells. A mass shift at the 12th lysine residue of histone H4 is consistent with lactylation. The data were plotted as the means ± standard deviations. Two-tailed Student’s t test in (**B**), *P < 0.05.

IHC staining analysis of lactylation modifications in hepatocellular carcinoma (HCC) tissues revealed significant differences between malignant and adjacent non-neoplastic tissues (Fig. 1A, B). Interestingly, IHC assessment demonstrated markedly elevated pan-Kla levels in HCC specimens compared to matched normal controls, with particularly pronounced nuclear localization (Fig. 1A). Nuclear pan-Kla intensity was increased in 84.7% of primary HCC cases (Fig. 1C). Among metastatic HCC cases, 64% showed higher nuclear pan-Kla intensity in metastatic lesions compared to their corresponding primary tumors (Fig. S1A). These progressive increases in lactylation modification strongly correlate with the metastasis and progression of HCC. Patients exhibiting elevated nuclear pan-Kla levels demonstrated significantly poorer overall survival (Fig. 1D) and higher recurrence rates (Fig. 1E). Clinicopathological analysis revealed that the proportion of patients with high nuclear pan-Kla levels was significantly greater in advanced-stage (III-IV) HCC compared to those with early-stage (I-II) disease (Fig. S1B). Notably, among sorafenib-treated patients, high nuclear pan-Kla levels correlated with worse clinical outcomes (HR 2.005 vs 1.485; Fig. 1F) and substantially lower treatment response rates (90.9% resistance in high nuclear pan-Kla vs. 34.4% resistance in low nuclear pan-Kla group; Fig. 1G). These findings suggest nuclear lactylation accumulation might serve as: (i) a molecular hallmark of aggressive HCC phenotypes, and (ii) a predictive biomarker for sorafenib treatment resistance.

Lactate is the fundamental substrate for lactylation modifications. Nala (sodium lactate) ionizes in solution and releases lactate anions, which also act as a key substrate for lactylation. As expected, experimental modulation of lactate availability through exogenous supplementation or Nala treatment significantly altered pan-Kla levels (Fig. S1C, D). These results confirmed that changes in lactate anions level directly affect the overall lactylation pattern. We subsequently reviewed the existing literature and obtained data on lactylation in HCC tissues [19, 20]. However, tissue samples contain a mixture of tumor cells, immune cells, and other stromal components, making it difficult to determine which proteins in tumor cells carry lactylation marks. To clarify this and validate the lactylated protein residues identified in HCC tissues at the cellular level, we performed a 4D label-free quantitative lactylation proteome assay using the human HCC cell line (HCCLM3). Comprehensive 4D label-free proteomic profiling of HCCLM3 cells identified extensive lactylation alterations, including 1,584 hyperlactylated proteins (4,300 modification sites) and 278 hypolactylated proteins (334 sites) (Fig. S1E). Subcellular localization analysis demonstrated nuclear enrichment of lactylated proteins (47.9% of total, Fig. S1F). Enrichment analysis revealed significant enrichment of hyperlactylated proteins in critical oncogenic pathways including: (i) glycolytic metabolism, (ii) cell cycle regulation, (iii) transcriptional control, (iv) stemness maintenance, (v) apoptotic signaling, and (vi) histone modification (Fig. S1G). Lactylation motif analysis was performed by examining 10 flanking amino acids using Motif-X (MoMo), with differential specificity (DS) scores quantifying amino acid frequency variations. This analysis identified “xKxxxxxxVxKxxxxxxxxxx” (motif score = 23.86) as a characteristic lactylation signature in HCCLM3 cells (Fig. S1H). Comprehensive profiling revealed 21 distinct histone lactylation sites in HCCLM3 cells. Integrated analysis of published datasets identified the conservation of 6 histone lactylation sites across multiple cancer types, including HeLa cells (26 sites) [14], AGS cells (21 sites) [21], HCC tissues (26 and 11 sites) [19, 20], gastric cancer tissues (26 sites) [22], and colorectal cancer tissues (28 sites) [22] (Fig. 1H). Considering the aforementioned studies, histone lactylation on H3 and H4 may have a more critical role in activating gene expression than that on H2A and H2B [23]. Notably, Zhang et al. demonstrated that H4K12la exhibits a more robust response to hypoxia-driven elevations in lactate levels compared with H3K79la [14]. Additionally, H4K12la is preserved across species, as it has been detected not only in human samples but also in mice [24], piglets [25], and non-mammalian organisms including *Toxoplasma gondii* [26] and crustaceans [27]. This may be attributed to the evolutionary stability of the histone H4 sequence, particularly at the H4K12 site (Fig. S1I, J), as well as the core role of lactylation in linking cellular metabolism to epigenetic regulation. Another notable consideration is that among the 6 identified sites, only one antibody specific for H4K12la is currently commercially available. Based on these findings, our further study focused on the H4K12la. Mass spectrometry analysis of histone H4 in HCCLM3 cells confirmed the presence of lactylation at the 12th lysine residue, validating H4K12la occurrence (Fig. 1I).

### Increased levels of H4K12la in the nucleus are associated with poor prognosis in HCC patients and resistance to receptor tyrosine kinase inhibitor

**Fig. 2:**
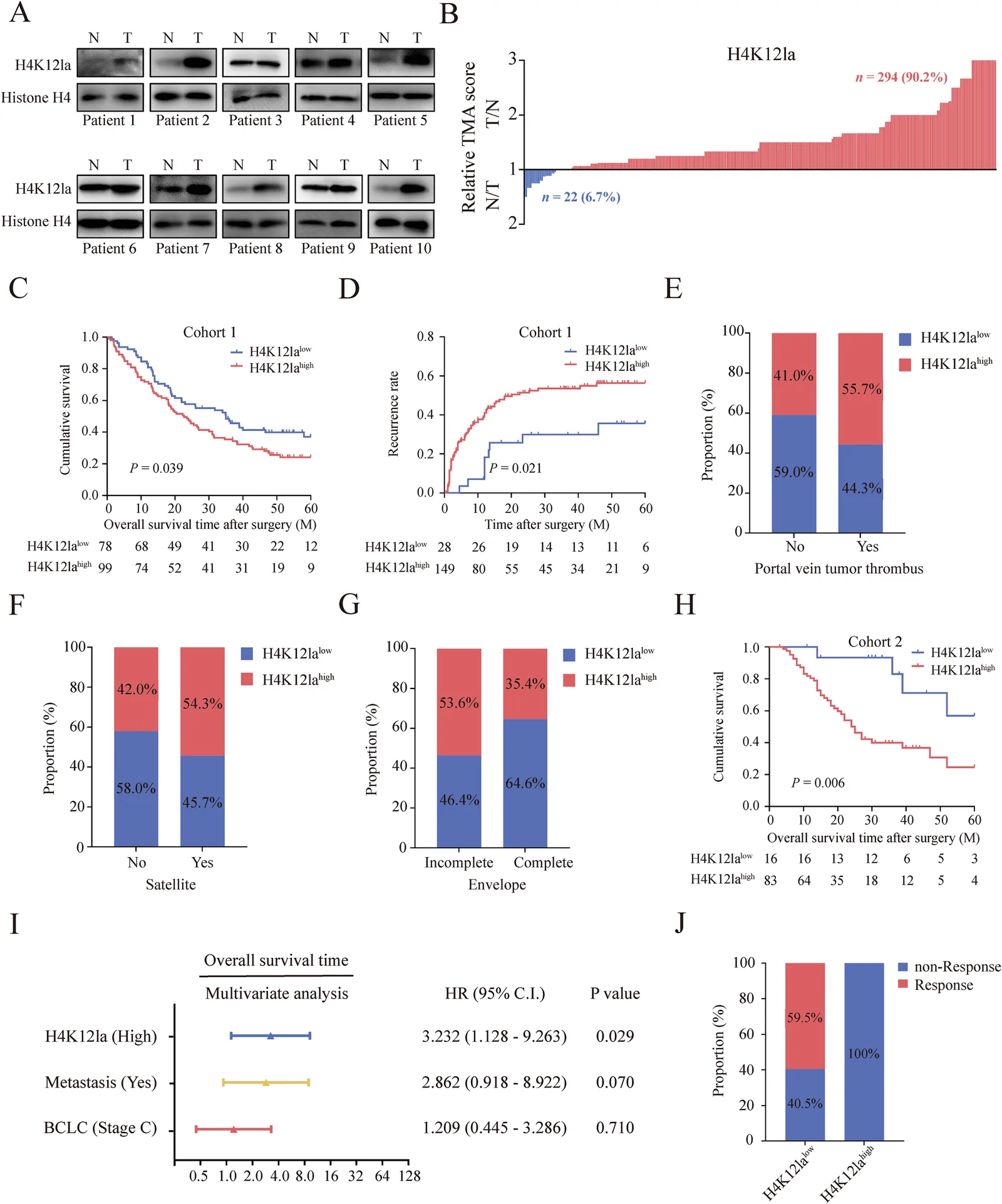
Increased levels of histone H4K12la are associated with poor prognosis and resistance to receptor tyrosine kinase inhibitor in HCC patients. **A** Western blot detection of histone H4K12la in HCC tumor (T) and normal liver (N) tissues. **B** IHC detection of H4K12la in HCC and normal liver tissues (PLC cohorts 1–3). A total of 90.2% of patients had higher H4K12la levels in HCC tissues (T/N > 1), and 6.7% of patients had higher H4K12la levels in normal tissues (N/T > 1). **C**, **D** Kaplan‒Meier survival analysis comparing overall survival (**C**) and disease-free survival (**D**) according to H4K12la levels in HCC patients (PLC cohort 1) analyzed by the log-rank test. **E**–**G** IHC detection of H4K12la levels in HCC tissues and analysis of their correlations with portal vein tumor thrombus status (**E**), tumor satellite lesion status (**F**), and tumor capsule integrity (**G**). **H** Kaplan‒Meier survival curves based on H4K12la levels in sorafenib-treated HCC patients (PLC cohort 2) analyzed by the log-rank test. **I** Multivariate Cox regression analysis for prognosis in sorafenib-treated HCC patients (PLC cohort 2). Three factors affecting the prognosis of HCC patients (H4K12la level, tumor metastasis status, and BCLC stage) were included. **J** Correlation between H4K12la levels and sorafenib efficacy in HCC patients. Disease progression (PD) indicates ineffective treatment; complete response (CR), partial response (PR), and stable disease (SD) indicate effective treatment.

Comparative analysis demonstrated significantly elevated H4K12la levels in HCC specimens relative to normal liver controls (Fig. 2A), with 90.2% of HCC cases exhibiting increased modification (Fig. 2B). Survival outcomes were markedly poorer in patients with high H4K12la level (Fig. 2C) and showed higher recurrence rates (Fig. 2D). Clinicopathological correlation studies revealed significant associations between elevated H4K12la and aggressive tumor features, including: (i) portal vein tumor thrombus, (ii) satellite lesion formation, and (iii) incomplete tumor encapsulation (Fig. 2E–G). These data suggested H4K12la may act as a molecular biomarker of invasive HCC phenotypes and prognostic index of patients.

Survival analysis of sorafenib-treated HCC patients revealed significantly worse outcomes in cases with elevated H4K12la levels (Fig. 2H). The prognostic impact of H4K12la exceeded that of pan-Kla modifications (HR = 2.63 vs 1.46), suggesting specific involvement in sorafenib resistance mechanisms. Multivariate analysis confirmed H4K12la as an independent predictor of poor sorafenib response (Fig. 2I). Strikingly, therapeutic response rates showed complete dichotomy between H4K12la-low (59.5% response) and H4K12la-high (0% response, Fig. 2J), implying H4K12la could be both a prognostic biomarker and potential mediator of sorafenib resistance in HCC.

### Lactate induces H4K12la and decreases the sensitivity of HCC cells to receptor tyrosine kinase inhibitors (sorafenib and lenvatinib)

**Fig. 3:**
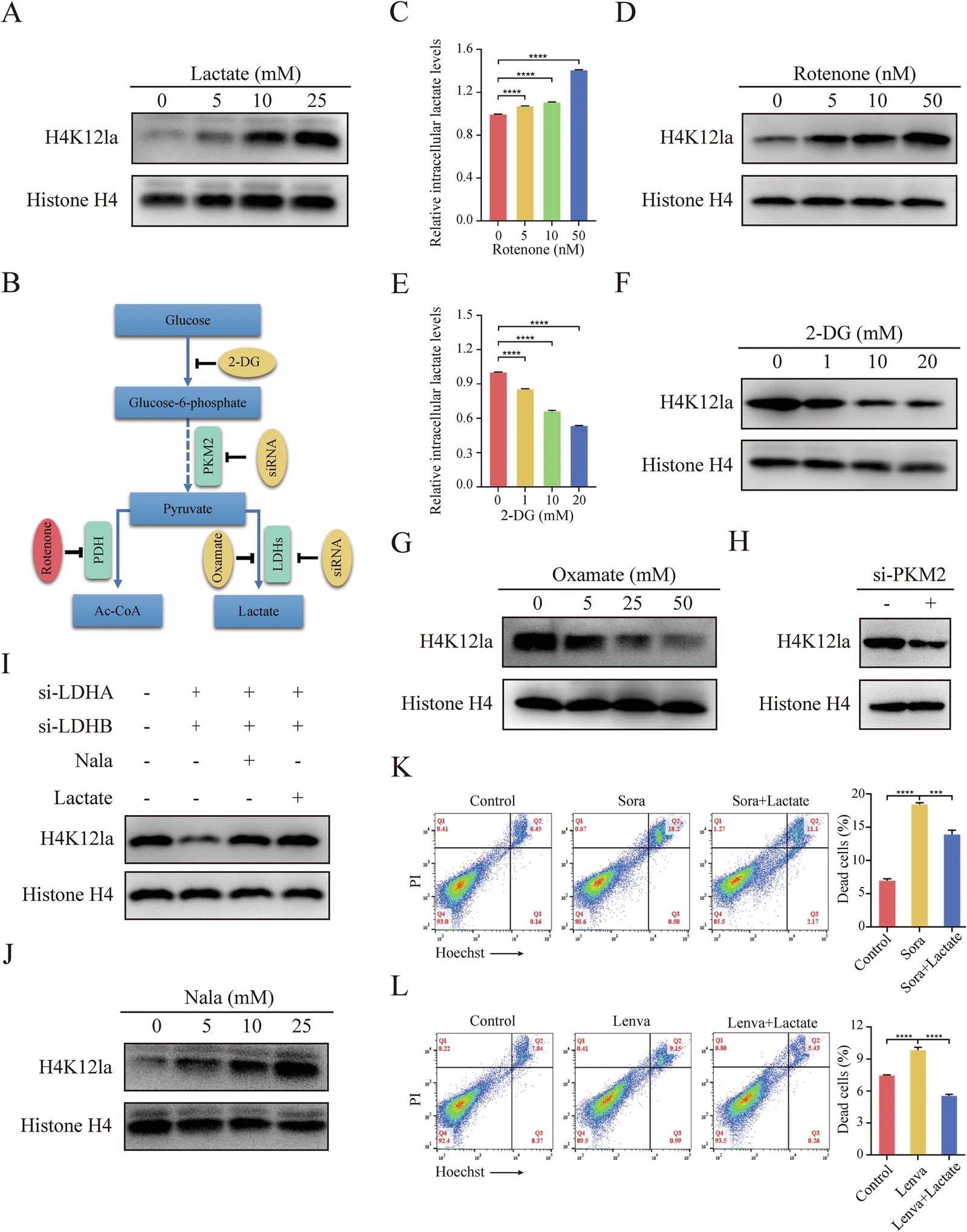
Lactate increases H4K12la levels and inhibits the sensitivity of HCC cells to receptor tyrosine kinase inhibitors (sorafenib or lenvatinib). **A** Western blot detection of H4K12la in HCCLM3 cells treated with exogenous lactate for 24 h. **B** Glycolytic pathway modulators regulating endogenous lactate production: 2-DG, oxamate, si-LDHs (LDHA/LDHB) and si-PKM2 suppress lactate generation, whereas rotenone increases it. **C**, **D** Intracellular lactate (**C**) and H4K12la (**D**) levels in HCCLM3 cells treated with rotenone for 24 h. **E**, **F** Intracellular lactate (**E**) and H4K12la (**F**) levels in HCCLM3 cells treated with 2-DG for 24 h. **G** Western blot detection of H4K12la in HCCLM3 cells treated with oxamate for 24 h. **H** Western blot detection of H4K12la in HCCLM3 cells after transfection of PKM2 siRNA. **I** Western blot detection of H4K12la in HCCLM3 cells after treatment with LDH siRNA and exogenous lactate or Nala. **J** Western blot detection of H4K12la in HCCLM3 cells treated with Nala for 24 h. **K**, **L** Flow cytometry was used to assess the effect of exogenous lactate treatment on HCCLM3 cell death induced by sorafenib (Sora) (**K**) or lenvatinib (Lenva) (**L**). An average of *n* = 3 independent replicates is shown. The data are presented as the means ± standard deviations. One-way ANOVA in (**C**, **E**, **K**, **L**) was used for the statistical analyses. ***P < 0.001 and ****P < 0.0001.

To determine whether alterations in intracellular lactate levels influence H4K12la in HCC cells, we experimentally manipulated lactate production and examined the subsequent changes in H4K12la modification. Initial observations revealed a dose- and time-dependent relationship between exogenous lactate exposure and H4K12la levels in HCCLM3 and HepG2 cells (Figs. 3A, S2A, S2B). Modulation of aerobic glycolysis through key metabolic enzymes significantly altered lactylation dynamics (Fig. 3B). Building on this, we employed both pharmacological and genetic strategies to either increase or decrease intracellular lactate. Rotenone, an inhibitor of mitochondrial electron transport chain Complex I, impairs mitochondrial respiration and thereby enhances anaerobic glycolysis, resulting in increased lactate production and enhanced lactylation. Herein, rotenone progressively increased both intracellular lactate concentrations and H4K12la levels (Figs. 3C, 3D, S2C, S2D), while 2-DG-mediated glycolysis suppression reduced lactate production and corresponding H4K12la deposition (Figs. 3E, 3F, S2E, S2F). Similarly, oxamate treatment (LDHA inhibition) demonstrated concentration-dependent reduction of H4K12la (Figs. 3G, S2G). Genetic confirmation was achieved through PKM2 knockdown, which significantly attenuated H4K12la levels (Figs. 3H, S2H, S2I), establishing glycolytic flux as a primary regulator of histone lactylation. Genetic silencing of lactate dehydrogenase isoforms (LDHA/B) via siRNA-mediated knockdown significantly reduced H4K12la levels in HCCLM3 and HepG2 cells (Figs. 3I, S2J-S2L). This effect was fully rescued by exogenous lactate or Nala supplementation (Figs. 3I, S2L). Elevation of lactylation through Nala treatment demonstrated dose-dependent increases in H4K12la (Figs. 3J, S2M). Exogenous lactate preconditioning significantly attenuated sorafenib- and lenvatinib-induced cell death in HCCLM3 and HepG2 cells, as quantified by flow cytometric analysis (Figs. 3K, L, S2N, O).

### AARS1 and HDAC11 function as the primary writer and eraser of H4K12la in HCC cells

**Fig. 4:**
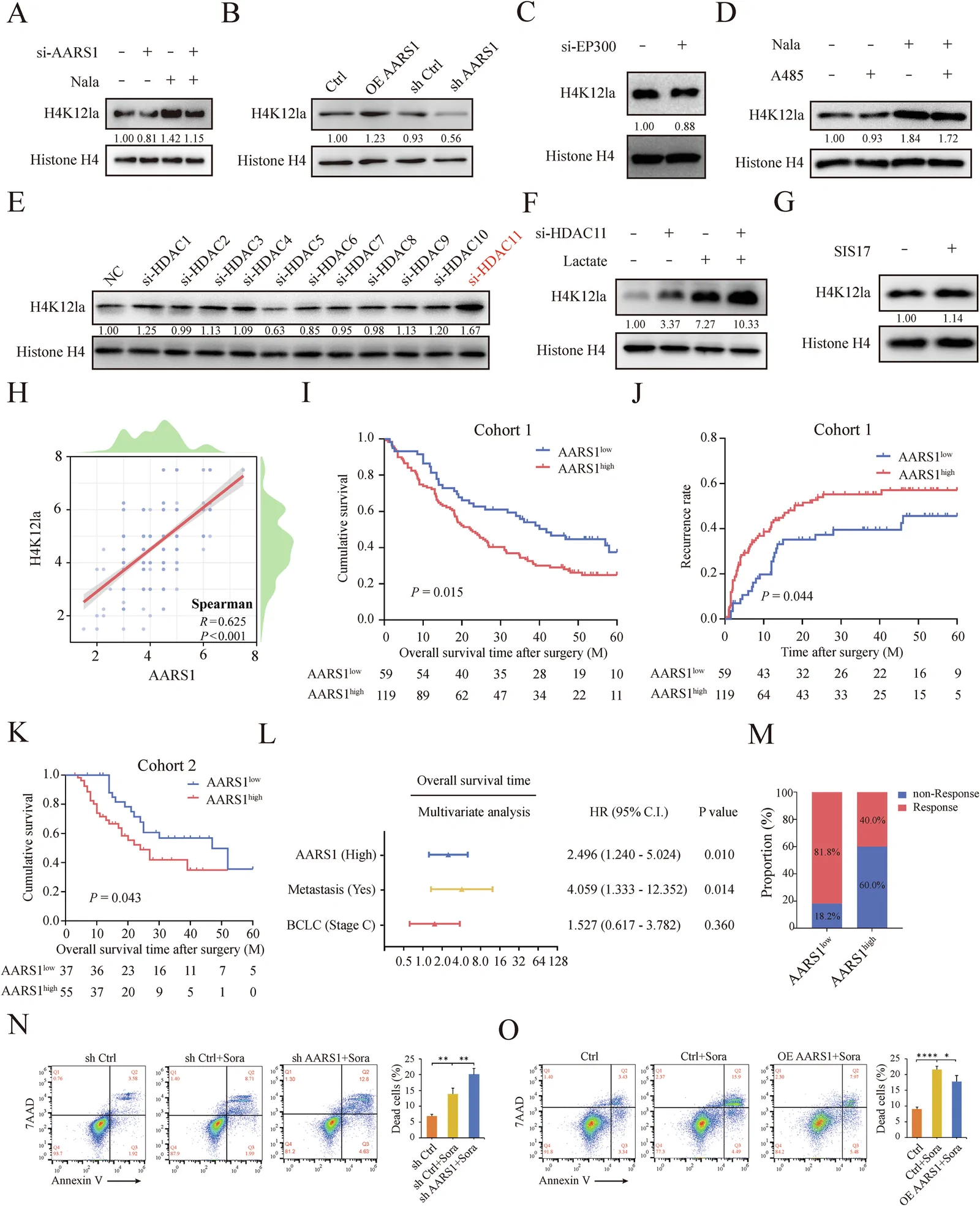
The increase in H4K12la modification by AARS1 suppresses the sensitivity of HCC to sorafenib. **A** Western blot detection of H4K12la in HCCLM3 cells after AARS1 siRNA and Nala treatment. **B** Western blot detection of H4K12la in HCCLM3 cells with AARS1 overexpression or knockdown. **C** Western blot of H4K12la in HCCLM3 cells after EP300 siRNA interference treatment. **D** Western blot of H4K12la in HCCLM3 cells after P300 inhibitor A-485 and Nala treatment. **E** Western blot of H4K12la in HCCLM3 cells after HDAC1-11 siRNA interference treatment. **F** Western blot of H4K12la in HCCLM3 cells after HDAC11 siRNA and lactate treatment. **G** Western blot of H4K12la in HCCLM3 cells after HDAC11 inhibitor SIS17 treatment. **H** Spearman correlation analysis of H4K12la and AARS1 scores in HCC tissues from PLC cohort 1-3. **I**, **J** Kaplan‒Meier survival analysis comparing overall survival (**I**) and disease-free survival (**J**) based on AARS1 expression in HCC patients (PLC cohort 1) analyzed by the log-rank test. **K** Kaplan‒Meier survival analysis comparing overall survival based on AARS1 expression in patients with sorafenib-treated HCC (PLC cohort 2) analyzed by the log-rank test. **L** Multivariate Cox regression for prognosis (OS) in the sorafenib cohort (PLC cohort 2) with AARS1 expression level, tumor metastasis status, and BCLC stage. **M** Correlation of AARS1 expression and sorafenib efficacy in HCC patients. Disease progression (PD) indicates ineffective treatment; complete response (CR), partial response (PR), and stable disease (SD) indicate effective treatment. **N**–**O** Flow cytometry analysis of the effects of AARS1 knockdown (**N**) or overexpression (**O**) on the death of HCCLM3 cells induced by sorafenib (Sora). Densitometric quantification of Western blot bands was performed using ImageJ software, followed by normalization to the corresponding loading control. An average of *n* = 3 independent replicates is shown. The data are presented as the means ± standard deviations. One-way ANOVA in (**N**, **O**) was used for the statistical analyses, *P < 0.05, **P < 0.01, ****P < 0.0001.

AARS1 and p300 have been identified as putative lactyltransferases [14, 16, 28]. siRNA-mediated AARS1 knockdown in HCC cells (HCCLM3 and HepG2) markedly reduced H4K12la levels regardless of Nala treatment (Figs. 4A, S3A). Consistent with this, stable AARS1 overexpression elevated H4K12la, while AARS1 knockdown diminished it (Fig. 4B). In contrast, p300 suppression via either siRNA interference (Figs. 4C, S3B) or pharmacological inhibition (A-485) (Fig. 4D) only marginally decreased H4K12la levels. These findings position AARS1 as the primary mediator of H4K12la, with p300 playing a secondary role. We next sought to identify the delactylase responsible for H4K12la removal. Histone deacetylases (HDACs) are key enzymes that regulate protein deacetylation [29]. Systematic evaluation of HDAC1-11 revealed that class I (HDAC1/3), class II (HDAC4), and class IV (HDAC11) HDACs modulated H4K12la levels without exogenous lactate (Fig. 4E). Among these, HDAC11 exhibited the most pronounced regulatory effect on H4K12la (Fig. 4E), suggesting its predominant role as a H4K12 delactylase in HCC cells (HCCLM3). Subsequently, we evaluated HDAC11 protein expression in tumor tissues from sorafenib-treated HCC patients (PLC cohort 2) using IHC staining. Spearman correlation analysis revealed a negative correlation between HDAC11 and H4K12la levels in HCC (Fig. S3C; *R* = − 0.310, *P* = 0.036). Further evidence confirmed that HDAC11 acts as a H4K12la delactylase in HCC: (i) elevated H4K12la following HDAC11 knockdown in HepG2 cells (Fig. S3D), and (ii) elevated H4K12la in HCCLM3 cells upon either HDAC11 interference (Fig. 4F) or pharmacological inhibition (SIS17 treatment; Fig. 4G). Together, these results establish AARS1 and HDAC11 as the principal opposing regulators of H4K12la dynamics in HCC, functioning as the dominant lactyltransferase and delactylase, respectively.

### AARS1 induces resistance of HCC cells to sorafenib by increasing H4K12 lactylation levels

The clinical relevance of the AARS1-H4K12la axis was evaluated in three HCC cohorts (PLC cohorts 1–3). PLC Cohorts 1 and 3 included pathologically confirmed PLC patients undergoing curative surgical resection, and PLC Cohort 2 included PLC patients receiving sorafenib therapy. AARS1 and H4K12la expression in HCC tissues from three PLC cohorts was assessed by IHC. IHC staining was scored based on intensity and proportion, and the final score was calculated as their product. AARS1 expression positively correlated with H4K12la levels (Fig. 4H) and was overexpressed in 76.7% of HCC tissues versus normal liver (Fig. S3E). High AARS1 expression correlated with worse overall survival (Fig. 4I), increased recurrence risk (Fig. 4J), and aggressive clinicopathological features, including incomplete tumor encapsulation and multifocal lesions (Fig. S3F, G). In sorafenib-treated patients, high AARS1 expression predicted poorer prognosis (HR 2.631 vs. 1.458; Fig. 4K) and was independently prognostic (Fig. 4L). Treatment response analysis showed an 81.8% response rate in low-AARS1 patients versus 60.0% nonresponse in high-AARS1 patients (Fig. 4M). Flow cytometry analysis confirmed AARS1 knockdown enhanced sorafenib-induced cell death, while overexpression conferred resistance (Fig. 4N, O), directly linking AARS1 to drug sensitivity. These findings establish AARS1 as both a prognostic biomarker and a critical modulator of targeted drug response in HCC.

### RAPGEF3-RAP1 signaling is downstream pathway of H4K12la and correlates with receptor tyrosine kinase inhibitor resistance

**Fig. 5:**
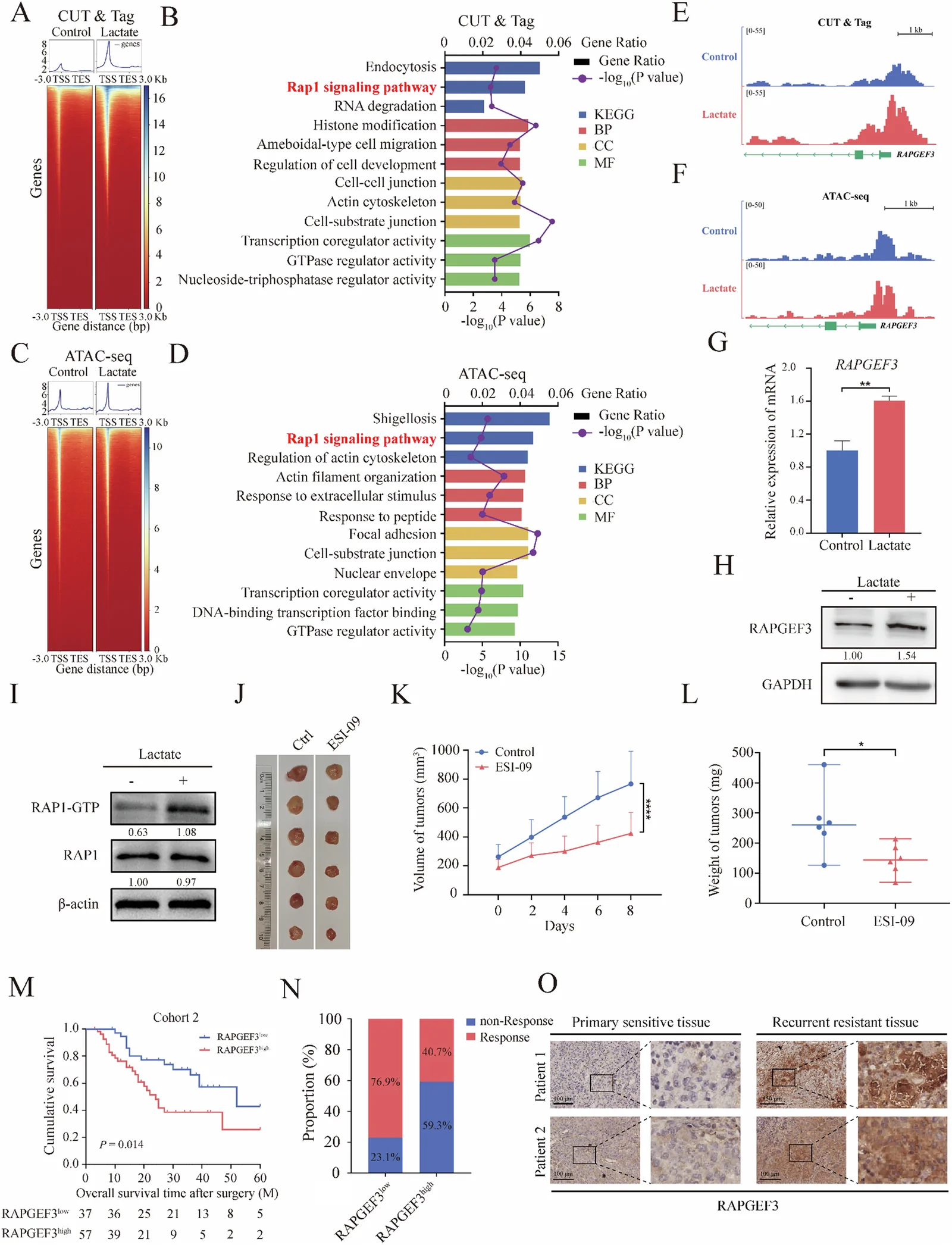
RAPGEF3-RAP1 signaling axis is downstream pathway of H4K12la and correlates with receptor tyrosine kinase inhibitor resistance. **A** The CUT&Tag assay revealed H4K12la changes in HCCLM3 cell promoters upon treatment with exogenous lactate, which is visualized with deepTools. The heatmap shows differences in the binding peaks. **B** GO and KEGG analyses of genes with differential H4K12la peak increases in HCCLM3 cell promoters, assessing biological processes (BP), cellular components (CC), and molecular functions (MF). **C** ATAC-seq was used to detect chromatin accessibility changes in HCCLM3 cell promoters upon treatment with exogenous lactate; the results were visualized using deepTools. The heatmap shows peak differences. **D** GO and KEGG analyses of genes with increased chromatin accessibility in HCCLM3 cell promoters, assessing BPs, CCs, and MFs. **E**, **F** CUT&Tag (**E**) and ATAC-seq (**F**) assays were used to detect H4K12la binding and chromatin accessibility changes in the *RAPGEF3* promoter in HCCLM3 cells treated with exogenous lactate via the *Integrative Genomics Viewer* (*IGV*). **G**, **H** RT‒qPCR (**G**) and Western blot (**H**) were used to detect RAPGEF3 expression changes in HCCLM3 cells treated with exogenous lactate. **I** Western blot analysis of RAP1 activity changes in HCCLM3 cells treated with exogenous lactate. **J**–**L** Nude mouse experiments were performed to assess the effect of the RAPGEF3 inhibitor ESI-09 on HCC growth, and the tumor volume (**J&K**) and weight (**L**) were compared (*n* = 6 for each group). **M** Kaplan‒Meier curves were used to analyze survival based on RAPGEF3 expression in sorafenib-treated HCC patients (PLC cohort 2) by the log-rank test. **N** Correlation analysis links RAPGEF3 expression in HCC to sorafenib efficacy (χ^2^ = 4.607, *P* = 0.032), with disease progression (PD) considered as ineffective treatment and complete response (CR), partial response (PR), and stable disease (SD) considered as effective treatment. **O** RAPGEF3 expression was compared between sorafenib-resistant recurrent HCC tissues and their primary sensitive counterparts from the same patients. Densitometric quantification of Western blot bands was performed using ImageJ software, followed by normalization to the corresponding loading control. The data are presented as the means ± standard deviations, Two-way ANOVA in (**K**) was used for the statistical analyses, *P < 0.05, **P < 0.01, ****P < 0.0001 .

CUT&Tag assays using an anti-H4K12la antibody identified H4K12la-regulated genes in HCCLM3 cells. Lactate treatment significantly enhanced H4K12la signal density near transcription start sites (TSSs) (Fig. 5A), with 2,727 genes exhibiting increased promoter binding and 809 showing decreased binding. H4K12la peaks were predominantly localized to promoters (66.4%) compared to introns (16.8%) and intergenic regions (12.6%), while decreased peaks displayed distinct distributions (34.8% promoters, 40.2% introns) (Fig. S4A). Functional enrichment analysis revealed that genes with elevated H4K12la binding were associated with endocytosis, RAP1 signaling, apoptosis, RNA degradation, and histone modification (Fig. 5B). Notably, RAP1 signaling enrichment, which governs cell adhesion, migration, and survival, highlights its central role in the H4K12la regulatory network in HCC. Furthermore, ATAC-seq analysis of exogenous lactate-treated HCCLM3 cells demonstrated increased chromatin accessibility around TSSs (Fig. 5C), with 2,925 gene promoters showing enhanced accessibility and 830 exhibiting reduced accessibility. Increased accessibility peaks were distributed across promoters (36.6%), introns (34.2%), and distal intergenic regions (23.6%), while decreased peaks followed a similar pattern (37.1% promoters, 33.2% introns, 24.4% intergenic) (Fig. S4B). Genes with heightened promoter accessibility were also enriched in the RAP1 signaling pathway (Fig. 5D). Subsequently, we conducted an integrated analysis of the CUT&Tag and ATAC-seq datasets and identified 483 commonly upregulated genes (Fig. S4C). More importantly, KEGG pathway analysis revealed significant enrichment of the RAP1 signaling pathway (Fig. S4D). Collectively, these findings demonstrate that lactate-induced upregulation of H4K12la transcriptionally modulates RAP1 pathway genes, thereby mediating its biological effects in HCC.

Within the RAP1 signaling pathway, RAPGEF3 (also known as EPAC1) and RAPGEF4 (also known as EPAC2) serve as critical activators [23]. CUT&Tag analysis demonstrated enhanced H4K12la binding at both *RAPGEF3* and *RAPGEF4* promoters following lactate treatment (Figs. 5E, S4E), while ATAC-seq revealed increased chromatin accessibility exclusively at the *RAPGEF3* locus (Figs. 5F, S4F). Consistent with these observations, lactate selectively induced RAPGEF3 expression (Figs. 5G, H, S4G, S4H) and activated RAP1 signaling (Fig. 5I). ESI-09 is characterized as an inhibitor of RAPGEF3 with favorable pharmacological and toxicological properties, and has shown promising application prospects in various fields, including cancer [30–32]. Herein, ESI-09 treatment significantly suppressed tumor progression in vivo, leading to decreased tumor volume (Fig. 5J, K) and reduced tumor weight (Fig. 5L). Clinical specimens exhibited RAPGEF3 overexpression in 80.0% of HCC cases compared to normal liver tissues (Fig. S4I), with lower expression observed in tumors with complete capsules (Fig. S4J). Sorafenib-treated patients with elevated RAPGEF3 expression demonstrated poorer survival outcomes (Fig. 5M) and reduced treatment response (59.3% non-responders vs. 76.9% responders in low expressers; Fig. 5N). Upregulated RAPGEF3 expression was observed in sorafenib-resistant recurrent HCC compared to their primary sensitive tumor tissues (Fig. 5O), further suggesting its role in targeted drug resistance.

### Targeting AARS1-H4K12la-RAPGEF3 axis overcomes targeted drug resistance in HCC

**Fig. 6:**
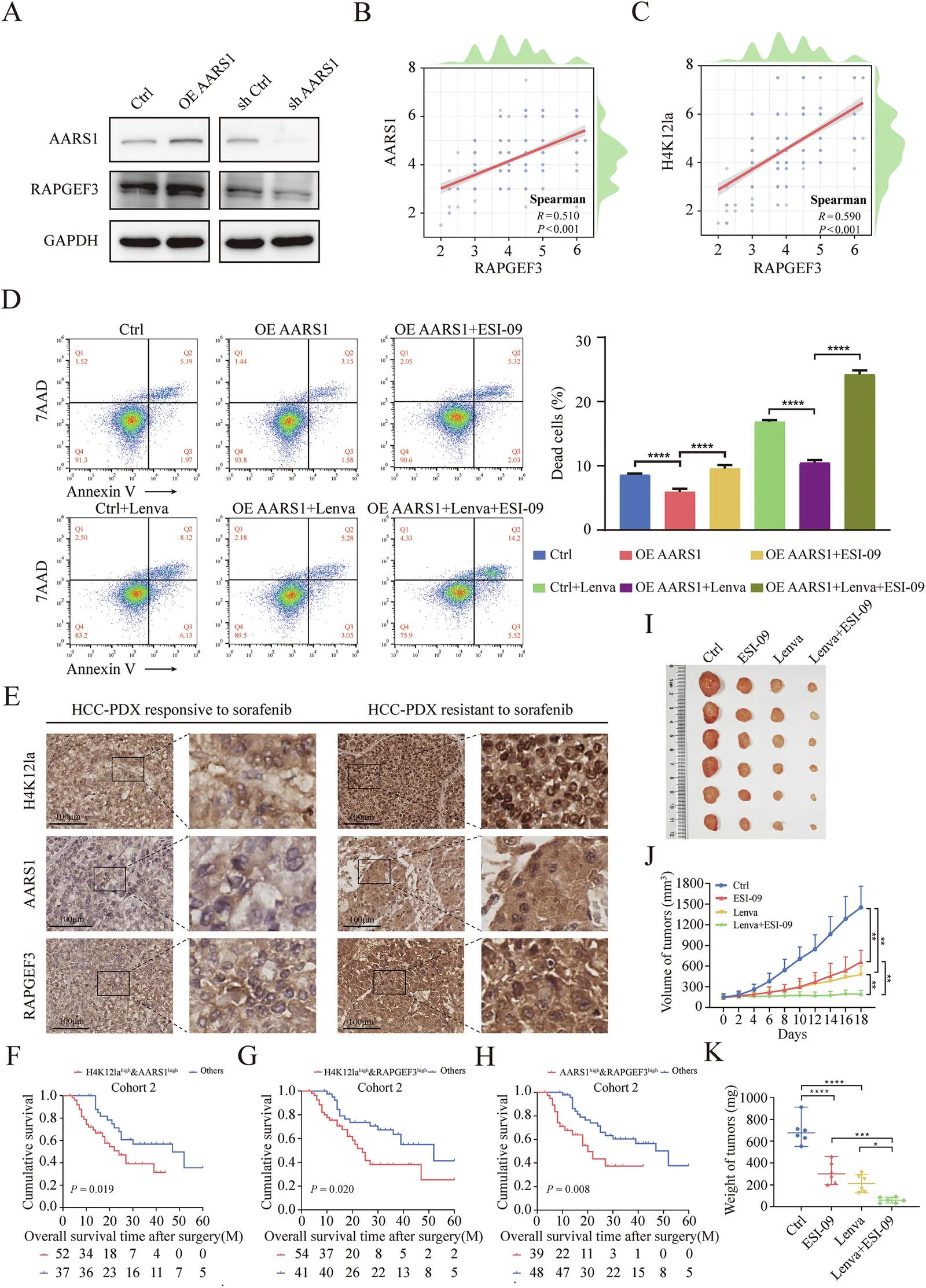
The AARS1-H4K12la-RAPGEF3 axis drives targeted drug resistance and poor prognosis in HCC. **A** Western blot showing RAPGEF3 changes in HCCLM3 cells with AARS1 overexpression or knockdown. **B**, **C** Correlations of AARS1 (**B**) and H4K12la (**C**) scores with RAPGEF3 score were assessed in HCC tumor tissues (PLC cohorts 1‒3) by Spearman analysis. **D** Flow cytometry was used to detect the effect of the RAPGEF3 inhibitor ESI-09 on lenvatinib-induced death in AARS1-overexpressing HCCLM3 cells. **E** AARS1 and RAPGEF3 expression and H4K12la levels were compared in patient-derived xenografts (PDXs) from sorafenib-sensitive and sorafenib-resistant HCC patients. **F**–**H** Kaplan‒Meier curves were used to analyze survival on the basis of H4K12la and AARS1 (**F**), AARS1 and RAPGEF3 (**G**), or H4K12la and RAPGEF3 (**H**) levels in patients with HCC (PLC cohort 2) by the log-rank test (OS). **I**–**K** Nude mouse experiments were performed to test the effects of ESI-09 and/or lenvatinib on HCC growth, and the tumor volume (**I**, **J**) and weight (**K**) were compared (*n* = 6 for each group). The data are presented as means ± standard deviations, One-way ANOVA in (**D**, **K**) and Two-way ANOVA in (**J**) were used for the statistical analyses, *P < 0.05, **P < 0.01, ***P < 0.001, and ****P < 0.0001.

The regulatory relationship between AARS1 and RAPGEF3 was firstly investigated using HCCLM3 cell lines with stable AARS1 overexpression or knockdown. AARS1 overexpression elevated RAPGEF3 expression, while AARS1 knockdown suppressed it (Fig. 6A). Clinical correlation analysis of HCC patient samples (PLC cohorts 1–3) demonstrated positive associations between AARS1 and RAPGEF3 expression (Fig. 6B) and between H4K12la levels and RAPGEF3 expression (Fig. 6C), supporting AARS1-mediated upregulation of RAPGEF3 through H4K12la. Pharmacological inhibition of RAPGEF3 with ESI-09 reversed AARS1-induced suppression of cell death and restored receptor tyrosine kinase inhibitor sensitivity (Figs. 6D, S5), confirming the role of RAPGEF3 in AARS1-mediated receptor tyrosine kinase inhibitor resistance.

Comparative analysis of HCC tissues from sorafenib-responsive and nonresponsive patients revealed significantly elevated AARS1 and RAPGEF3 expression, along with increased H4K12la levels in nonresponders (Fig. S6). This pattern was validated in patient-derived xenograft (PDX) models, with sorafenib-resistant tissues demonstrating higher H4K12la levels and elevated AARS1/RAPGEF3 expression compared to sensitive controls (Fig. 6E). Kaplan-Meier survival analysis of sorafenib-treated HCC patients identified three high-risk profiles: (i) High AARS1 with high H4K12la (Fig. 6F); (ii) High RAPGEF3 with high H4K12la (Fig. 6G); (iii) High AARS1 with high RAPGEF3 (Fig. 6H). These results establish the AARS1/H4K12la/RAPGEF3 axis as a predictive index for sorafenib response and patient stratification. Pharmacological inhibition of RAPGEF3 with ESI-09 significantly increased the sensitivity of HCC cells to sorafenib (Fig. S7A) and lenvatinib (Fig. S7B). This observation was further validated using HJC0197, another inhibitor of RAPGEF3, which similarly increased the efficacy of both drugs in vitro (Fig. S7C, D). Consistently, application of RAPGEF3 inhibitor notably enhanced the therapeutical effect of lenvatinib on HCC growth (Fig. 6I–K). These findings suggest that targeting RAPGEF3 function may serve as a promising strategy to overcome targeted drug resistance in HCC.

## Discussion

Although molecular receptor tyrosine kinase inhibitor has exhibited superior efficacy in hepatocellular carcinoma (HCC) treatment, targeted drug resistance constitutes a major clinical challenge [33], underscoring the urgent need to delineate its molecular basis and identify reliable predictive biomarkers. This study identifies histone H4K12la as a critical epigenetic driver of HCC targeted drug resistance. The AARS1-H4K12la-RAPGEF3 axis links metabolic reprogramming to drug resistance through activation of RAP1 signaling, thereby nominating RAPGEF3 as a potential therapeutic target to overcome resistance. These findings provide an integrated molecular framework connecting metabolic alterations, epigenetic dysregulation, and treatment resistance in HCC.

Histone lactylation has emerged as a critical epigenetic regulator of tumor progression across multiple cancer types. In renal cell carcinoma, VHL inactivation establishes an H3K18 lactylation-PDGFRβ feedback loop that drives tumor growth [34]. Ocular melanoma utilizes H3K18 lactylation to upregulate YTHDF2, leading to degradation of tumor suppressors PER1 and TP53 [35]. In HCC, histone lactylation has been identified as a central mechanism governing tumor progression through multiple modification-specific pathways. H3K18 lactylation exhibits multifaceted oncogenic effects, including promoting ferroptosis resistance after microwave ablation through NFS1 activation [36], mediating metabolic reprogramming through a SRSF10/glycolysis/H3K18la positive feedback loop [37]. The H3K56 lactylation modification plays an essential role in maintaining liver cancer stem cell stemness and tumorigenic potential [38, 39]. These findings collectively position histone lactylation as a master regulator of HCC malignant progression. The current study, for the first time, demonstrates that H4K12la is significantly elevated in HCC tissues and correlates strongly with poor clinical outcomes, including reduced survival, increased recurrence, and decreased sensitivity to targeted drugs. The clarification of H4K12 lactylation and its function add new concept to the regulatory network through which histone modification influences tumor malignance.

The dynamic regulation of protein lactylation involves opposing activities of lactyltransferases (writers) and delactylases (erasers). While p300 was initially characterized as a lactyltransferase utilizing L-lactyl-CoA [14]. Its biological relevance in cancer remains questionable due to the extremely low physiological concentrations of lactyl-CoA - approximately 1,000-fold lower than acetyl-CoA levels [15]. This limitation of lactyl-CoA-dependent mechanisms prompted investigation of alternative pathways, leading to the pivotal discovery of AARS1 as a lactyltransferase operating through a lactyl-CoA-independent mechanism [16, 28, 40]. We found that AARS1 directly utilizes lactate as a substrate, exhibiting high binding affinity comparable to its canonical substrate alanine. Given the characteristic lactate accumulation in the tumor microenvironment, AARS1 probably represents the predominant physiological driver of lactylation. This enzymatic function appears conserved across malignancies, where AARS1-mediated lactylation promotes oncogenesis through diverse pathways: in gastric cancer, nuclear-translocated AARS1 lactylates and activates YAP-TEAD to establish a proliferative feedforward loop [16]; it also modifies p53, disrupting its phase separation and DNA binding capacity to facilitate tumor progression [28]; AARS1-mediated YTHDC1 lactylation reinforces enfortumab vedotin resistance in bladder cancer [40]. Our current findings reveal AARS1 as the primary regulator of histone H4K12 lactylation, with clinical data demonstrating that high AARS1 expression correlates with reduced treatment response and serves as an independent prognostic factor in sorafenib-treated HCC patients. The dynamic nature of lactylation requires not only lactylation mechanism but also balanced delactylation machinery. In general, protein deacylation modification is usually catalyzed by HDAC family members (classes I-IV) and SIRTs (1-7) [41]. Emerging evidence has implicated HDAC family members (class I) catalyze histone delactylation and SIRT (1-3) catalyze non-histone proteins [12, 29, 42]. Our study firstly reported class IV HDAC (HDAC11) could catalyze histone delactylation and identified HDAC11 as a key regulator of H4K12 delactylation in HCC cells. Together, these findings establish AARS1 and HDAC11 as opposing regulators of H4K12la levels in HCC, providing both mechanistic insights into lactylation dynamics and potential therapeutic avenues for targeting this oncogenic modification.

Under the regulation of RAPGEFs, inactive RAP1 (bound to GDP) binds to GTP, thereby being activated. Subsequently, the downstream pathways of PI3K/AKT and BRAF/MEK/ERK are activated [43–45]. As a key modulator of cellular signaling, RAP1 exerts profound effects on cancer cell survival, proliferation, and drug resistance [46]. As multikinase inhibitors, sorafenib or lenvatinib targets receptor tyrosine kinases (VEGFR, PDGFR, FGFR, c-Kit, Ret, and FLT3, etc.) and inhibits their downstream PI3K/AKT and BRAF/MEK/ERK pathways, suppressing the survival and proliferation of HCC cells. Our study demonstrates that H4K12la enhances RAPGEF3 expression by increasing its promoter accessibility, leading to the activation of RAP1 signaling, which compromises the inhibition of tyrosine kinases by targeted drugs. To our knowledge, this is the first evidence regarding histone lactylation could mediate targeted drug resistance in cancer. Moreover, our in vitro and in vivo data reveals the synergistic therapeutic effects when combining the RAPGEF3 inhibitor with lenvatinib, suggesting RAPGEF3 might serve as an interventional target to overcome targeted drug resistance in HCC. This is worthy of further investigation by clinical trials.

Our study also presents several thought-provoking findings worthy of in-depth discussion. In CUT&Tag assays performed with an H4K12la-specific antibody in HCC cells, we observed that lactate treatment generally elevated H4K12la enrichment at most gene promoter regions. Intriguingly, a subset of gene promoters exhibited an unexpected reduction in H4K12la signal abundance. A plausible biological explanation for this phenomenon is that lactylation is a dynamic epigenetic modification jointly regulated by a variety of writers and erasers. The reduced H4K12la levels at specific promoters may serve as a secondary or indirect regulatory effect, which could result from the crosstalk among distinct histone modifications and the interference of competitive signaling pathways at the H4K12 residue. Given that gene transcription is orchestrated by multiple histone modifications across distinct amino acid residues, rather than being solely governed by H4K12la, this moderate overlap still indicates a concordance between H4K12la deposition and chromatin accessibility at these promoters. Of note, KEGG pathway enrichment analysis of these 483 overlapping genes revealed significant enrichment of the RAP1 signaling pathway, further confirming the regulatory effect of H4K12la on the core gene expression of the RAP1 signaling axis. In addition, we identified 2727 genes with elevated H4K12la signals in promoter regions and 2925 genes with increased chromatin accessibility in HCC cells, among which 483 overlapping genes were obtained. Given that gene transcription is coordinately regulated by multiple histone modifications at distinct histone residues, rather than being solely dominated by H4K12la, this moderate degree of gene overlap still indicates a consistent correlation between H4K12la deposition and chromatin accessibility at the promoters of these genes. Notably, KEGG pathway enrichment analysis of these 483 overlapping genes demonstrated that the RAP1 signaling pathway exhibited the most significant enrichment, further supporting that lactate‑induced H4K12la upregulation predominantly modulates the expression of genes involved in the RAP1 signaling pathway.

Admittedly, the present study also has certain limitations. First, although the downstream target genes in RAP1 pathway transcriptionally regulated by H4K12la—identified through integrated CUT&Tag and ATAC-seq analyses—have been validated by in vitro experiments. However, the absence of RNA-seq data restricts our further systematic and comprehensive profiling of the global transcriptional landscape governed by H4K12la in HCC, which will be addressed in our future research. Second, the therapeutic potential of dual RTK and RAPGEF3 inhibition was examined solely in subcutaneous xenograft models. The lack of available sorafenib-resistant HCC clinical specimens precluded the generation of resistant xenografts. Evaluating the synergistic efficacy of this combinatorial strategy in sorafenib-resistant settings thus remains a critical objective of our ongoing research.

## Conclusions

This study demonstrates that elevated H4K12la serves as an independent prognostic biomarker associated with poor response to sorafenib in HCC. We identified AARS1 as a lactyltransferase and HDAC11 as a delactylase that dynamically regulate H4K12la. Mechanistically, AARS1-catalyzed H4K12la promotes RAPGEF3 transcription and activates RAP1, enabling the activation of PI3K/AKT and BRAF/MEK/ERK signaling pathways, which collectively undermine the efficacy of tyrosine kinase inhibitors. Importantly, pharmacological inhibition of RAPGEF3 synergizes with lenvatinib to suppress HCC growth, highlighting its therapeutic potential. Collectively, our study advances the understanding of lactylation in HCC pathogenesis by: (i) demonstrating a higher level of protein lactylation in the nucleus than in the cytoplasm of HCC cells; (ii) identifying AARS1 as a histone lactyltransferase and HDAC11 as a delactylase; (iii) establishing AARS1-H4K12la-RAPGEF3 axis as a potential biomarker for targeted therapy; and (iv) proposing RAPGEF3 as a potential interventional target to overcome targeted drug resistance in HCC. These findings provide a molecular framework for lactylation-targeted strategies to improve HCC treatment, offering new perspectives to overcome targeted drug resistance—an issue of urgent clinical importance.

## Supplementary information

Supplementary Material

Supplementary Material-Uncropped gels for Western Blots

## Data Availability

Data are available under the auspices of the principal investigator upon reasonable request.
