# Supplementary material for: AARS1-catalyzed H4K12 lactylation promotes HCC resistance to targeted therapy by activating RAPGEF3-RAP1 signaling
Source: Cell Death Dis. 2026 Jul 21;17(1):888. doi: 10.1038/s41419-026-09011-6 (PMC13672797; doi:10.1038/s41419-026-09011-6)
Supplement: Supplementary file 1 [file 41419_2026_9011_MOESM1_ESM.pdf]

Supplementary Material

Lactate-Induced H4K12 Lactylation via AARS1 Modulates HCC Sensitivity to Targeted Therapy by Activating RAPGEF3-RAP1 Signaling

Tanlun Zeng[1, 2, #], Wanwan Zhu[1, #], Guanqun Sun[1, #], Tengjiao Wang[3], Chenna Liu[1], Guanghui Hu[4], Junyu Liu[5], Pengyun Ling[6], Li Wang[1], Chang Fan[1], Terence Kin Wah Lee[7], Jianru Xiao[8, *], Xijun Liang[1, *], and Jin Ding[1, *]

[1]Clinical Cancer Institute, Center for Translational Medicine, Naval Medical University, Shanghai, China;

[2]National Key Laboratory of Immunity and Inflammation, Institute of Immunology, Naval Medical University, Shanghai, China;

[3]Center for Translational Medicine, Naval Medical University, Shanghai, China;

[4]Department of Plastic and Reconstructive Surgery, Shanghai Fourth People's Hospital, School of Medicine, Tongji University, Shanghai 200434, China;

[5]Department of Gastroenterology, The Third Affiliated Hospital of Sun Yat-Sen University, Guangzhou, China;

[6]Department of Blood Transfusion, Naval Specialty Medical Center, Naval Medical University, Shanghai, China;

[7]Department of Applied Biology and Chemical Technology, The Hong Kong Polytechnic University, Hong Kong, China;

[8]Institute of Orthopedics, Department of Orthopedic Oncology, Shanghai Changzheng Hospital, Naval Medical University, Shanghai, 200003, China.

[#] These authors contributed equally to this work.

Corresponding authors

Jin Ding, Clinical Cancer Institute, Center for Translational Medicine, Naval Medical University 800 Xiangyin Road, 200433 Shanghai, China. E-mail: dingjin1103@163.com.

Xijun Liang, Clinical Cancer Institute, Center for Translational Medicine, Naval Medical University, 800 Xiangyin Road, 200433 Shanghai, China. E-mail: liangxj_0711@163.com.

Jianru Xiao, Department of Orthopaedic Oncology, Changzheng Hospital, Naval Military Medical University, 415 Feng Yang Road, 200003 Shanghai, China. E-mail: jianruxiao83@163.com.

Supplementary Table S1. Clinicopathologic Features of 181 HCC Specimens in Cohort 1

**Characteristics**		**Number of patients**
Age (year)	≤50	98
	＞50	83
Gender	Male	146
	Female	35
HBsAg	Positive	141
	Negative	40
TNM stage	I~II	69
	III	22
AFP (ng/mL)	≤400	107
	＞400	73
Tumor size (cm)	≤5	66
	＞5	114
Tumor number	Single	144
	Multiple	37
Portal vein tumor thrombus	Yes	75
	No	104
Envelope	None	123
	Complete	55

Supplementary Table S2. Clinicopathologic Features of 101 HCC Specimens in Cohort 2

Characteristics		Number of patients
Age (year)	≤50	64
	＞50	37
Gender	Male	92
	Female	9
HBsAg	Positive	96
	Negative	5
BCLC stage	A~B	70
	C	29
Tumor size (cm)	≤5	57
	＞5	44
Tumor number	Single	53
	Multiple	48
Portal vein tumor thrombus	Yes	37
	No	57
Envelope	None	84
	Complete	12
Sorafenib efficacy	Non-Response	22
	Response	21

Supplementary Table S3. Clinicopathologic Features of 66 HCC Specimens in Cohort 3

**Characteristics**		**Number of patients**
Age (year)	≤50	39
	＞50	27
Gender	Male	57
	Female	9
HBsAg	Positive	56
	Negative	10
BCLC stage	A~B	32
	C	34
Tumor size (cm)	≤5	13
	＞5	53
Tumor number	Single	62
	Multiple	4
Portal vein tumor thrombus	Yes	55
	No	11
Envelope	None	38
	Complete	27

Supplementary Table S4. siRNA target sequences used in this study

Gene	Sequences (5’-3’)
H-HDAC1	CGGUUAGGUUGCUUCAAUC
H-HDAC2	CAGUCUCACCAAUUUCAGA
H-HDAC3	CAAGAGUCUUAAUGCCUUC
H-HDAC4	GCCAAAGAUGACUUCCCUC
H-HDAC5	CAGCAUGACCACCUGACAA
H-HDAC6	CGGUAAUGGAACUCAGCAC
H-HDAC7	CAAGUAGUUGGAACCAGAG
H-HDAC8	GCGUAUUCUCUACGUGGAU
H-HDAC9	GAGCAGUUAAUAGGCUUUA
H-HDAC10	CGGAGUCAGUGUGCAUGACAGUACA
H-HDAC11	CCCGACGUGGUGGUAUACA
H-PKM2	CAUCUACCACUUGCAAUUA
H-LDHA	GGCAAAGACUAUAAUGUAA
H-LDHB	GGGAAAGUCUCUGGCUGAU
H-AARS1	GGUGGAUGACAGCAGUGAAGA
H-EP300	GCACGAACUAGGAAAGAAA

Supplementary Table S5. Primers used in this study

Gene	Sequences (5'-3')
H-HDAC1-F	CTACTACGACGGGGATGTTGG
H-HDAC1-R	GAGTCATGCGGATTCGGTGAG
H-HDAC2-F	ATGGCGTACAGTCAAGGAGG
H-HDAC2-R	TGCGGATTCTATGAGGCTTCA
H-HDAC3-F	CCTGGCATTGACCCATAGCC
H-HDAC3-R	CTCTTGGTGAAGCCTTGCATA
H-HDAC4-F	GGCCCACCGGAATCTGAAC
H-HDAC4-R	GAACTCTGGTCAAGGGAACTG
H-HDAC5-F	GCATGAACTCTCCCAACGAGT
H-HDAC5-R	CCACTGTCACAGGGATGCTG
H-HDAC6-F	AAGAAGACCTAATCGTGGGACT
H-HDAC6-R	GCTGTGAACCAACATCAGCTC
H-HDAC7-F	GGCGGCCCTAGAAAGAACAG
H-HDAC7-R	CTTGGGCTTATAGCGCAGCTT
H-HDAC8-F	TCGCTGGTCCCGGTTTATATC
H-HDAC8-R	TACTGGCCCGTTTGGGGAT
H-HDAC9-F	AGTAGAGAGGCATCGCAGAGA
H-HDAC9-R	GGAGTGTCTTTCGTTGCTGAT
H-HDAC10-F	CTTGGGAAGCTCCTGTACCTC
H-HDAC10-R	ATGTTCAGCCACAGACTCCT
H-HDAC11-F	ACCCAGACAGGAGGAACCATA
H-HDAC11-R	TGATGTCCGCATAGGCACAG
H-PKM2-F	ATGTCGAAGCCCCATAGTGAA
H-PKM2-R	TGGGTGGTGAATCAATGTCCA
H-LDHA-F	TTCCGGATCTCATTGCCACG
H-LDHA-R	AAGGCTGCCATGTTGGAGAT
H-LDHB-F	GACGTGCGGGGTAGTACTTG
H-LDHB-R	TTGCCTCTTCTTCCGCAACT
H-AARS1-F	TCACCCAAGAGTTTGGCATTC
H-AARS1-R	CCTGGGAGGATTTTGGTGTCA
H-EP300-F	TGTGCCAAACCAGATGATGC
H-EP300-R	CAGGTTGAGCCAACTGTCCA
H-RAPGEF3-F	GACCGGAAGTACCACCTTAGG
H-RAPGEF3-R	AGATTCCCACAACTTGGCTCC
H-actin-F	TGGATCAGCAAGCAGGAGTA
H-actin-R	TCGGCCACATTGTGAACTTT

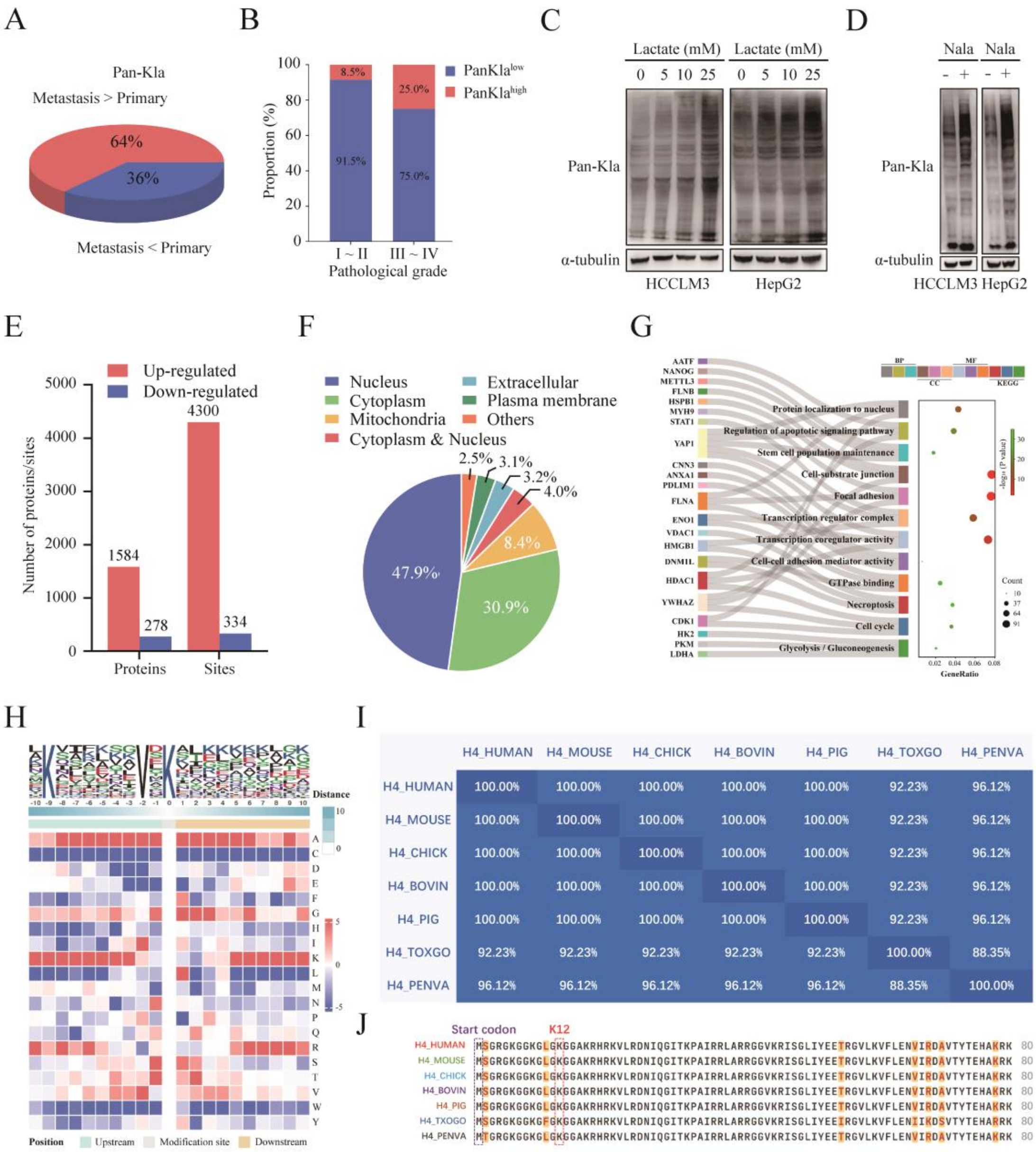

Figure S1. Proteomic analysis of lactylation modifications in HCC cells.

Figure S1. Proteomic analysis of lactylation modifications in HCC cells.

(A) Nuclear pan-Kla levels compared between primary and metastatic HCC foci. A total of 64% of patients had higher levels in metastatic foci, while 36% of patients had lower levels. (B) IHC detection of nuclear pan-Kla levels in primary HCC tissues and correlates with pathological grading. (C) Western blot detection of pan-Kla levels in HCC cell lines (HCCLM3, HepG2) treated with exogenous lactate for 24 h. (D) Western blot detection of pan-Kla levels in HCC cell lines (HCCLM3, HepG2) treated with 25 mM Nala for 24 h. (E) Quantitative statistics of lactylated proteins and sites in HCC cells. (F) Distribution of lactylated proteins in HCC cells: 796 (47.9%) in nucleus,

514 (30.9%) in cytoplasm, 139 (8.4%) in mitochondria, 66 (4.0%) in both nucleus and cytoplasm, 54 (3.2%) extracellular, 52 (3.1%) in plasma membrane. (G) GO and KEGG enrichment analyses were performed on proteins with elevated lactylation levels, including biological processe (BP), cellular component (CC), and molecular function (MF). (H) Motif characteristics of lactylation sites analyzed using MoMo tool. DS = -Log10 (p.value) × sign(diff.percent). Heatmap shows amino acid frequency changes around lactylation sites. (I) The sequence similarity of histone H4 among different species was compared. (J) The histone H4 sequences from *Homo sapiens* (human), *Mus musculus* (mouse), *Gallus gallus* (chick), *Bos taurus* (bovine), *Sus scrofa* (pig), *Toxoplasma gondii* (TOXGO) and *Penaeus vannamei* (PENVA) were subjected to sequence alignment.

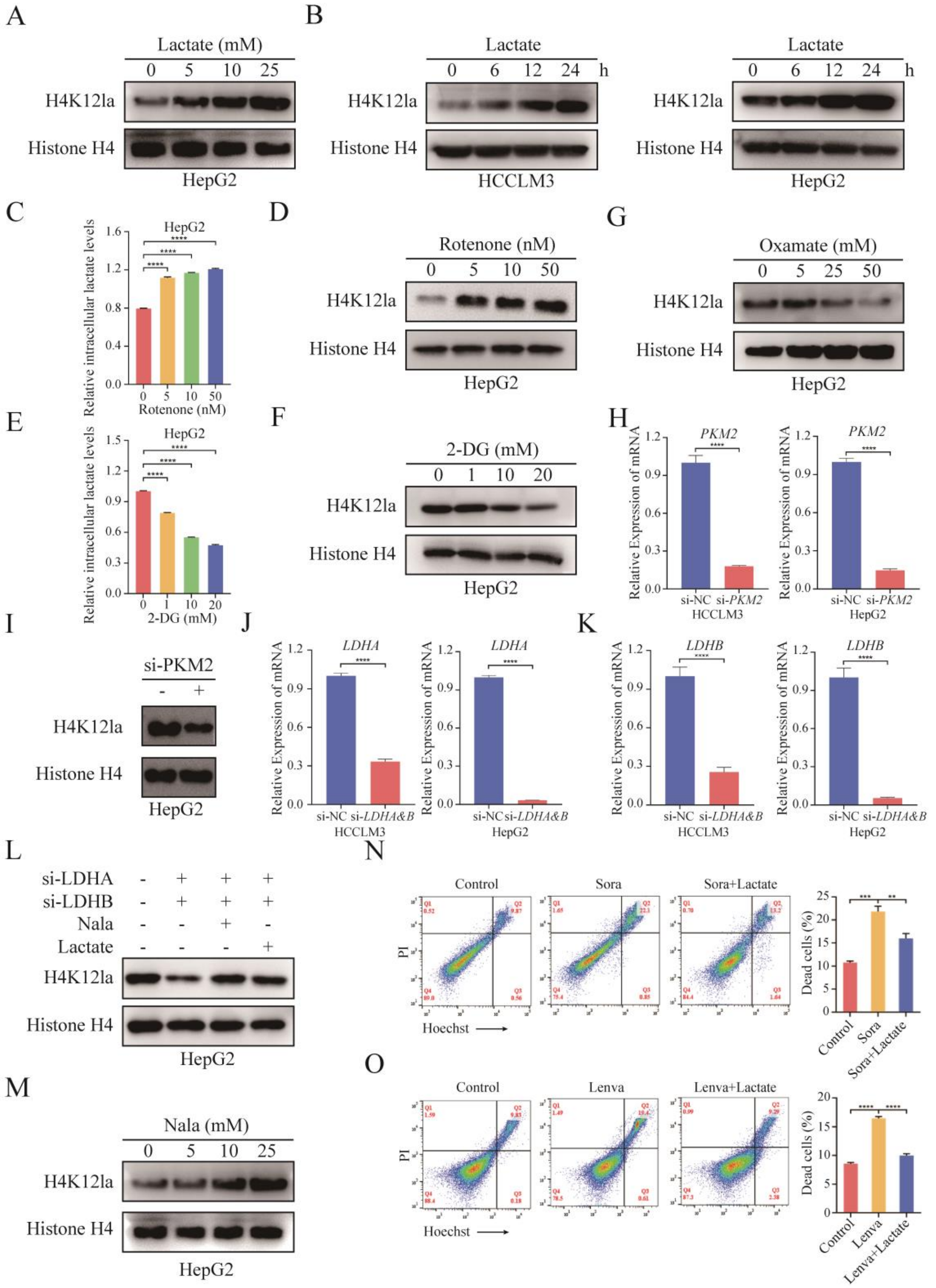

Figure S2. Lactate increases H4K12la levels to inhibit the sensitivity of HCC cells to receptor tyrosine kinase inhibitors (sorafenib or lenvatinib).

(A) Western blot detection of H4K12la in HepG2 cells with exogenous lactate for 24 h.

(B) Western blot detection of H4K12la in HCCLM3 or HepG2 cells with 25 mM lactate

at various times. (C-D) Intracellular lactate (C) and H4K12la (D) levels in HepG2 cells treated with Rotenone for 24 h. (E-F) Intracellular lactate (E) and H4K12la (F) levels in HepG2 cells treated with 2-DG for 24 h. (G) Western blot detection of H4K12la in HepG2 cells with oxamate for 24 h. (H) siRNA interference of *PKM2* expression in HCC cells (HCCLM3, HepG2), and RT-qPCR detection of interference efficiency. (I) Western blot detection of H4K12la changes in HepG2 cells after PKM2 siRNA interference. (J-K) siRNA interference of *LDHA* (J) or *LDHB* (K) expression in HCC cells (HCCLM3, HepG2), and RT-qPCR detection of interference efficiency. (L) Western blot detection of H4K12la changes in HepG2 cells after LDHs (LDHA&LDHB) siRNA interference and lactate or Nala treatment. (M) Western blot of H4K12la in HepG2 cells treated with Nala for 24 h. (N-O) Flow cytometry was used to detect the effect of exogenous lactate treatment on HepG2 cell death induced by sorafenib (Sora) (N) or lenvatinib (Lenva) (O). The data are presented as the means ± standard deviations, ** P<0.01, *** P<0.001, and **** P<0.0001.

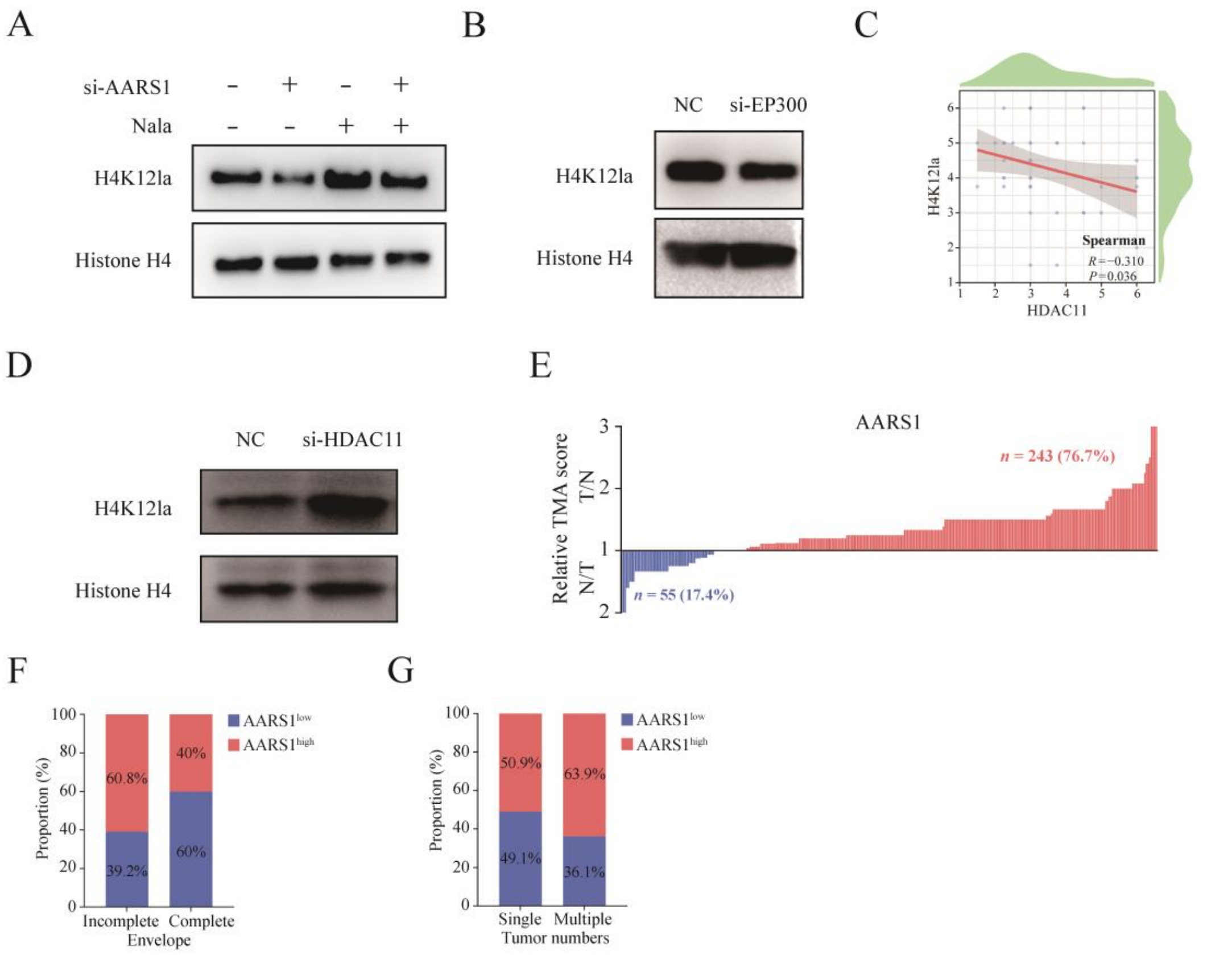

Figure S3. AARS1 and HDAC11 function as the primary writer and eraser of H4K12la in HCC.

(A) Western blot detection of H4K12la in HepG2 cells after AARS1 siRNA and Nala treatment. (B) Western blot detection of H4K12la in HepG2 cells after EP300 siRNA interference treatment. (C) Spearman correlation analysis of H4K12la and HDAC11 scores in HCC tissues from PLC cohort 2. (D)Western blot detection of H4K12la in HepG2 cells after HDAC11 siRNA interference treatment. (E) IHC detection of AARS1 expression in HCC and normal liver tissues (PLC cohorts 1–3). 76.7% had higher AARS1 in HCC (T/N>1); 17.4% had higher AARS1 in normal liver (N/T>1). (F) IHC detection of AARS1 expression in primary HCC tissues and analysis of their correlation with tumor capsule integrity. (G) IHC detection of AARS1 expression in primary HCC tissues and analysis of their correlation with tumor number.

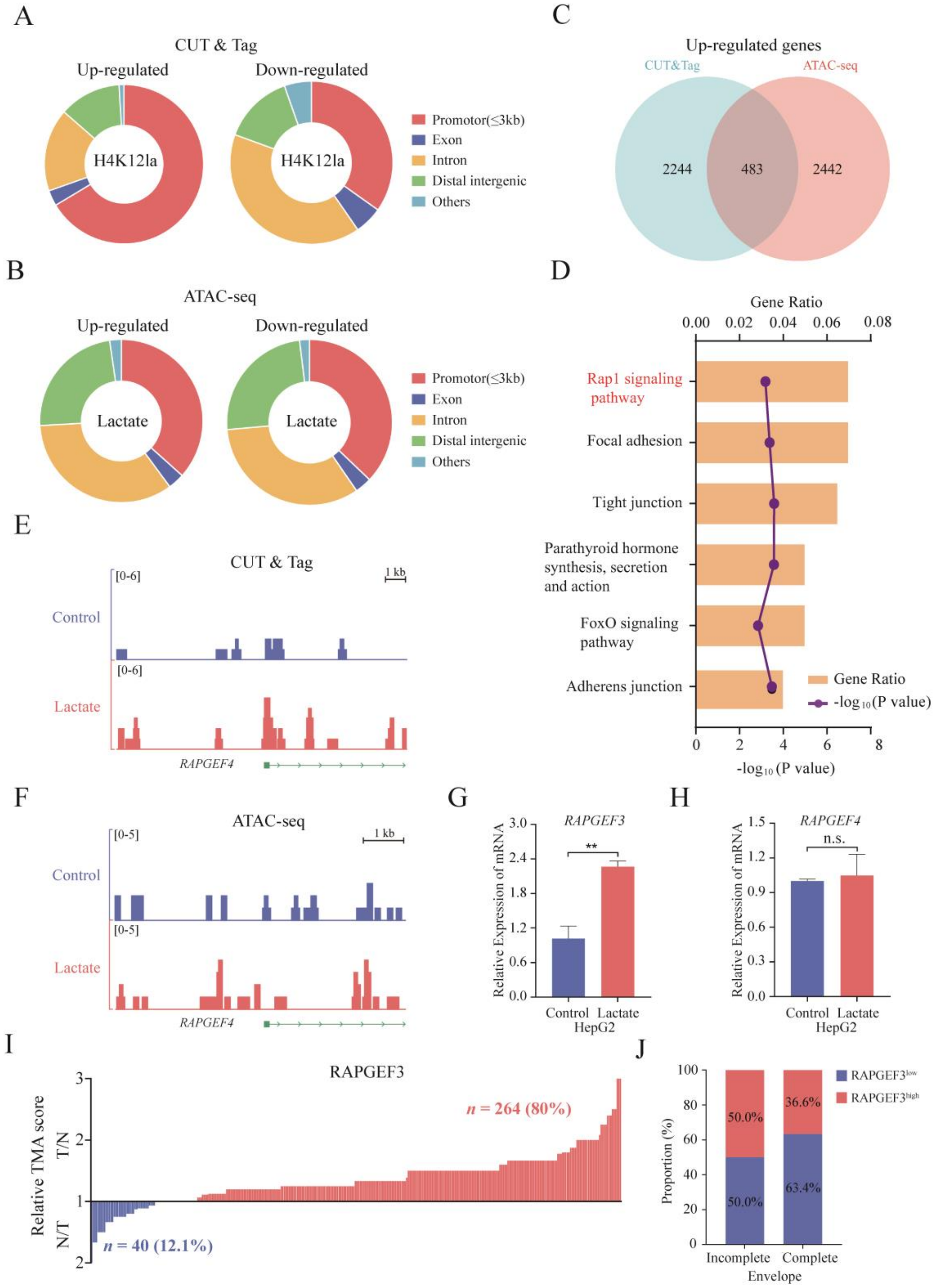

Figure S4. The RAPGEF3-RAP1 signaling pathway serves as a key downstream pathway of H4K12la.

(A) Genomic location analysis of differential peaks of H4K12la binding in HCC cells. For upregulated peaks, 66.4% are in promoters, 3.2% in exons, 16.8% in introns, and

12.6% in distal intergenic regions. For downregulated peaks, 34.8% are in promoters, 5.5% in exons, 40.2% in introns, and 14.0% in distal intergenic regions. (B) Genomic location analysis of differential peaks detected by ATAC-seq in HCC cells. For upregulated peaks, 36.6% are in promoters, 3.3% in exons, 34.2% in introns, and 23.6% in distal intergenic regions. For downregulated peaks, 37.1% are in promoters, 3.3% in exons, 33.2% in introns, and 24.4% in distal intergenic regions. (C) The Venn diagram illustrates the 483 commonly upregulated genes identified via integrated CUT&Tag and ATAC-seq analysis. (D) KEGG analyses of 483 commonly upregulated genes in CUT&Tag and ATAC-seq analysis. (E) CUT&Tag assays was used to detect H4K12la binding changes in RAPGEF4 promoter in HCC cells with exogenous lactate, viewed via Integrative Genomics Viewer (IGV). (F) ATAC-seq assays was used to detect peak changes in RAPGEF4 promoter in HCC cells with exogenous lactate, viewed via IGV. (G-H) RT-qPCR detection of expression changes in *RAPGEF3* (G) and *RAPGEF4* (H) in HepG2 cells with exogenous lactate. (I) IHC of RAPGEF3 in HCC and normal liver tissues (PLC cohorts 1-3): 80.0% higher in HCC (T/N>1), 12.1% higher in normal liver (N/T>1). (J) IHC of RAPGEF3 in primary HCC tissues and correlation with tumor capsule integrity. The data are presented as means ± standard deviations, ** $P<0.01$.

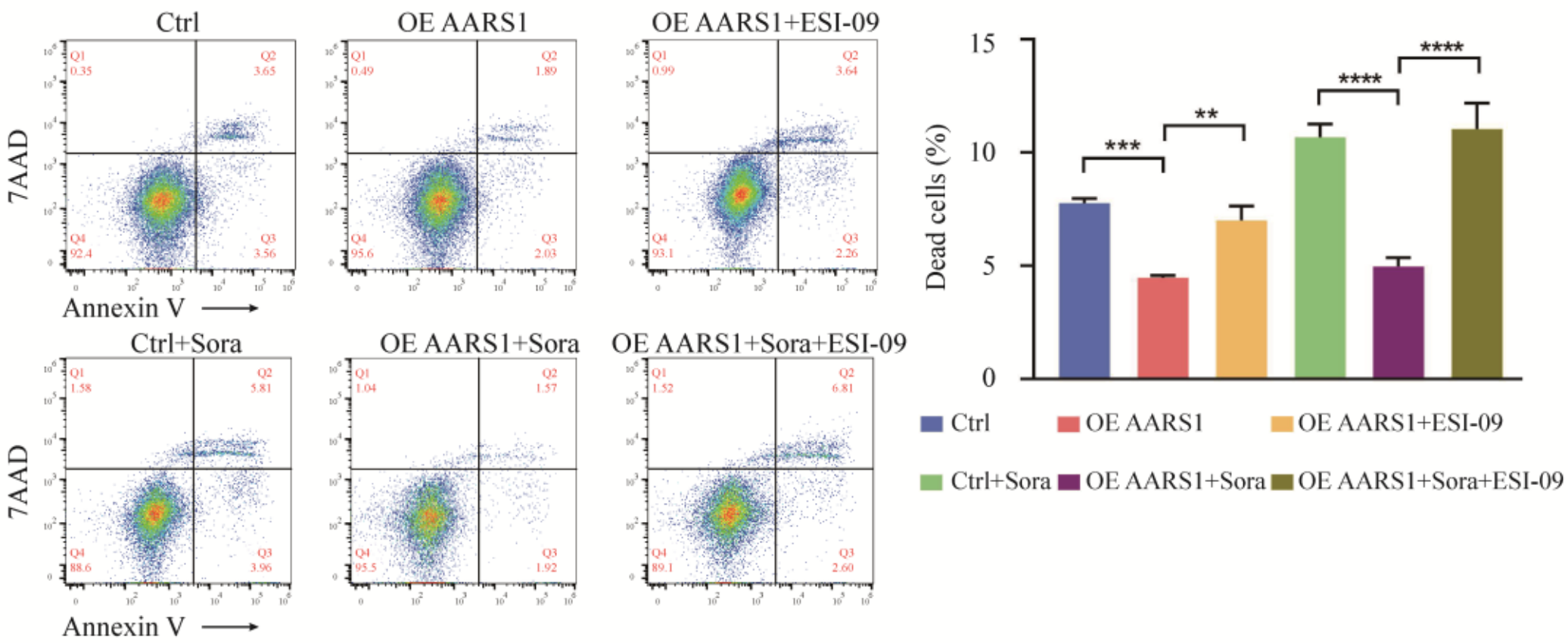

Figure S5. Inhibition of RAPGEF3 with ESI-09 reverses sorafenib-induced death in AARS1-overexpressing HCCLM3 cells.

Flow cytometry was used to detect the effect of the RAPGEF3 inhibitor ESI-09 on sorafenib-induced death in AARS1-overexpressing HCCLM3 cells. The data are presented as mans ± standard deviations, One-way ANOVA was used for the statistical analyses, ** P<0.01, *** P<0.001, and **** P<0.0001.

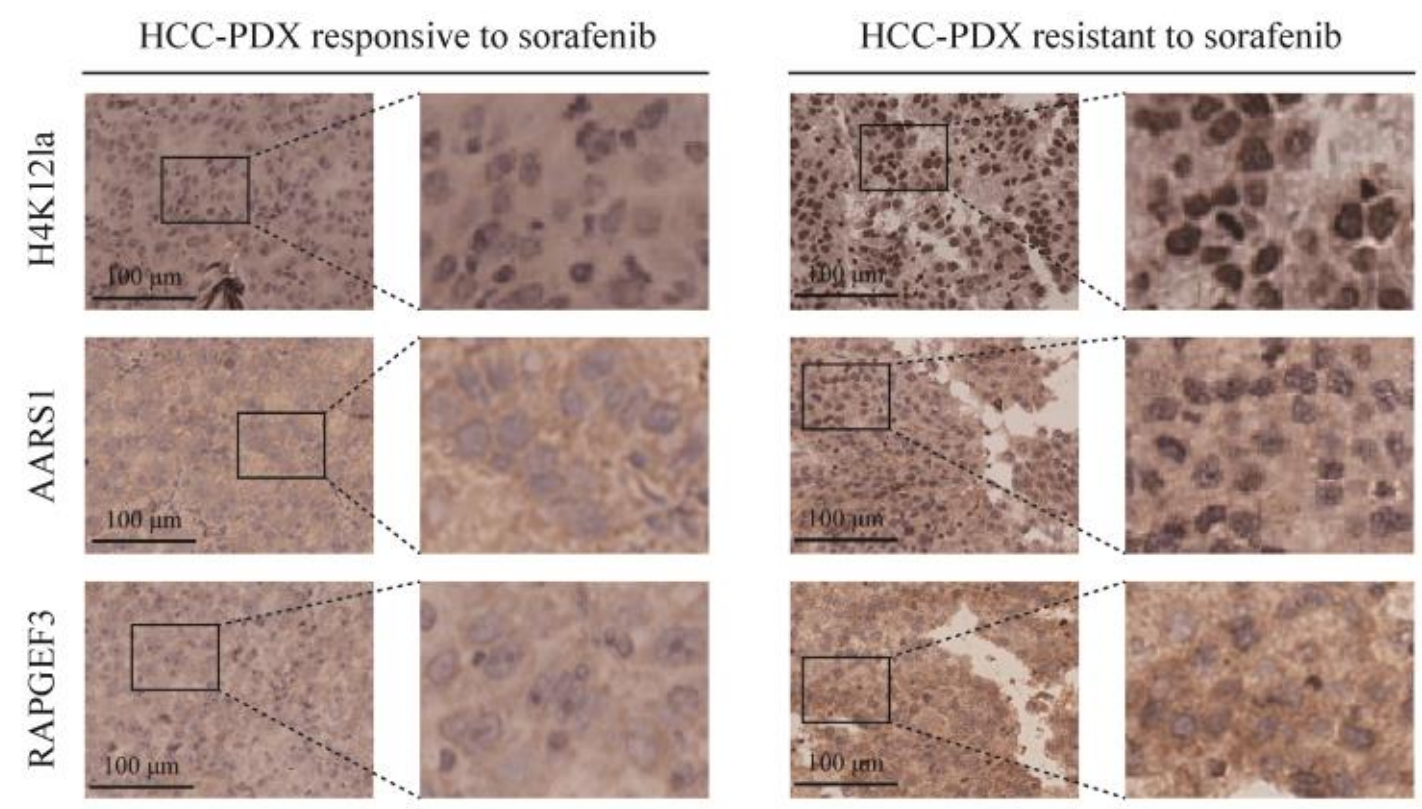

Figure S6. AARS1 and RAPGEF3 expression, along with H4K12la levels, are upregulated in sorafenib non-response HCC patients.

AARS1 and RAPGEF3 expression, along with H4K12la levels, were compared in HCC samples from sorafenib response and sorafenib non-response HCC patients.

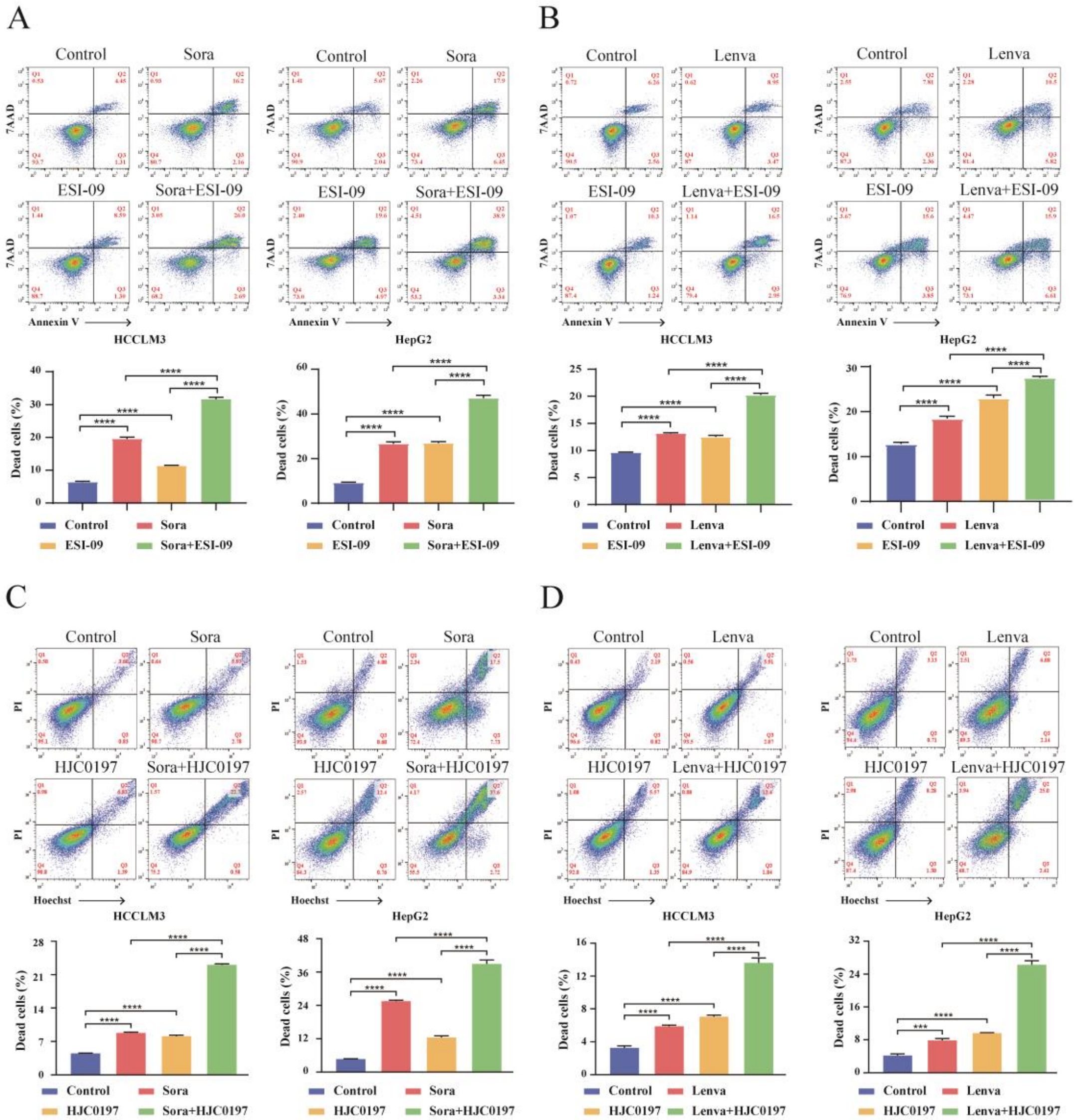

Figure S7. RAPGEF3 inhibition increases the sensitivity of receptor tyrosine kinase inhibitor efficacy in HCC cells.

(A) HCCLM3 and HepG2 cells were treated with sorafenib (Sora) or/and ESI-09, and changes in HCC cell death ratio were detected by flow cytometry. (B) HCCLM3 and HepG2 cells were treated with lenvatinib (Lenva) or/and ESI-09, and changes in HCC cell death ratio were detected by flow cytometry. (C) HCCLM3 and HepG2 cells were treated with sorafenib (Sora) or/and HJC0197, and changes in HCC cell death ratio were detected by flow cytometry. (D) HCCLM3 and HepG2 cells were treated with lenvatinib (Lenva) or/and HJC0197, and changes in HCC cell death ratio were detected by flow cytometry. The data are presented as means ±standard deviations, *** $P<0.001$,

and **** P<0.0001.
