# Supplementary material for: AARS1-catalyzed H4K12 lactylation promotes HCC resistance to targeted therapy by activating RAPGEF3-RAP1 signaling
Source: Cell Death Dis. 2026 Jul 21;17(1):888. doi: 10.1038/s41419-026-09011-6 (PMC13672797; doi:10.1038/s41419-026-09011-6)

Supplementary Material

Lactate-Induced H4K12 Lactylation via AARS1 Modulates HCC Sensitivity to Targeted Therapy by Activating RAPGEF3-RAP1 Signaling

Tanlun Zeng[1, 2, #], Wanwan Zhu[1, #], Guanqun Sun[1, #], Tengjiao Wang[3], Chenna Liu[1], Guanghui Hu[4], Junyu Liu[5], Pengyun Ling[6], Li Wang[1], Chang Fan[1], Terence Kin Wah Lee[7], Jianru Xiao[8, *], Xijun Liang[1, *], and Jin Ding[1, *]

[1]Clinical Cancer Institute, Center for Translational Medicine, Naval Medical University, Shanghai, China;

[2]National Key Laboratory of Immunity and Inflammation, Institute of Immunology, Naval Medical University, Shanghai, China;

[3]Center for Translational Medicine, Naval Medical University, Shanghai, China;

[4]Department of Plastic and Reconstructive Surgery, Shanghai Fourth People's Hospital, School of Medicine, Tongji University, Shanghai 200434, China;

[5]Department of Gastroenterology, The Third Affiliated Hospital of Sun Yat-Sen University, Guangzhou, China;

[6]Department of Blood Transfusion, Naval Specialty Medical Center, Naval Medical University, Shanghai, China;

[7]Department of Applied Biology and Chemical Technology, The Hong Kong Polytechnic University, Hong Kong, China;

[8]Institute of Orthopedics, Department of Orthopedic Oncology, Shanghai Changzheng Hospital, Naval Medical University, Shanghai, 200003, China.

[#] These authors contributed equally to this work.

Corresponding authors

Jin Ding, Clinical Cancer Institute, Center for Translational Medicine, Naval Medical University 800 Xiangyin Road, 200433 Shanghai, China. E-mail: dingjin1103@163.com.
Xijun Liang, Clinical Cancer Institute, Center for Translational Medicine, Naval Medical University, 800 Xiangyin Road, 200433 Shanghai, China. E-mail: liangxj_0711@163.com.
Jianru Xiao, Department of Orthopaedic Oncology, Changzheng Hospital, Naval Military Medical University, 415 Feng Yang Road, 200003 Shanghai, China. E-mail: jianruxiao83@163.com.

Uncropped gels for Western Blots in Figure 2

Figure 2A

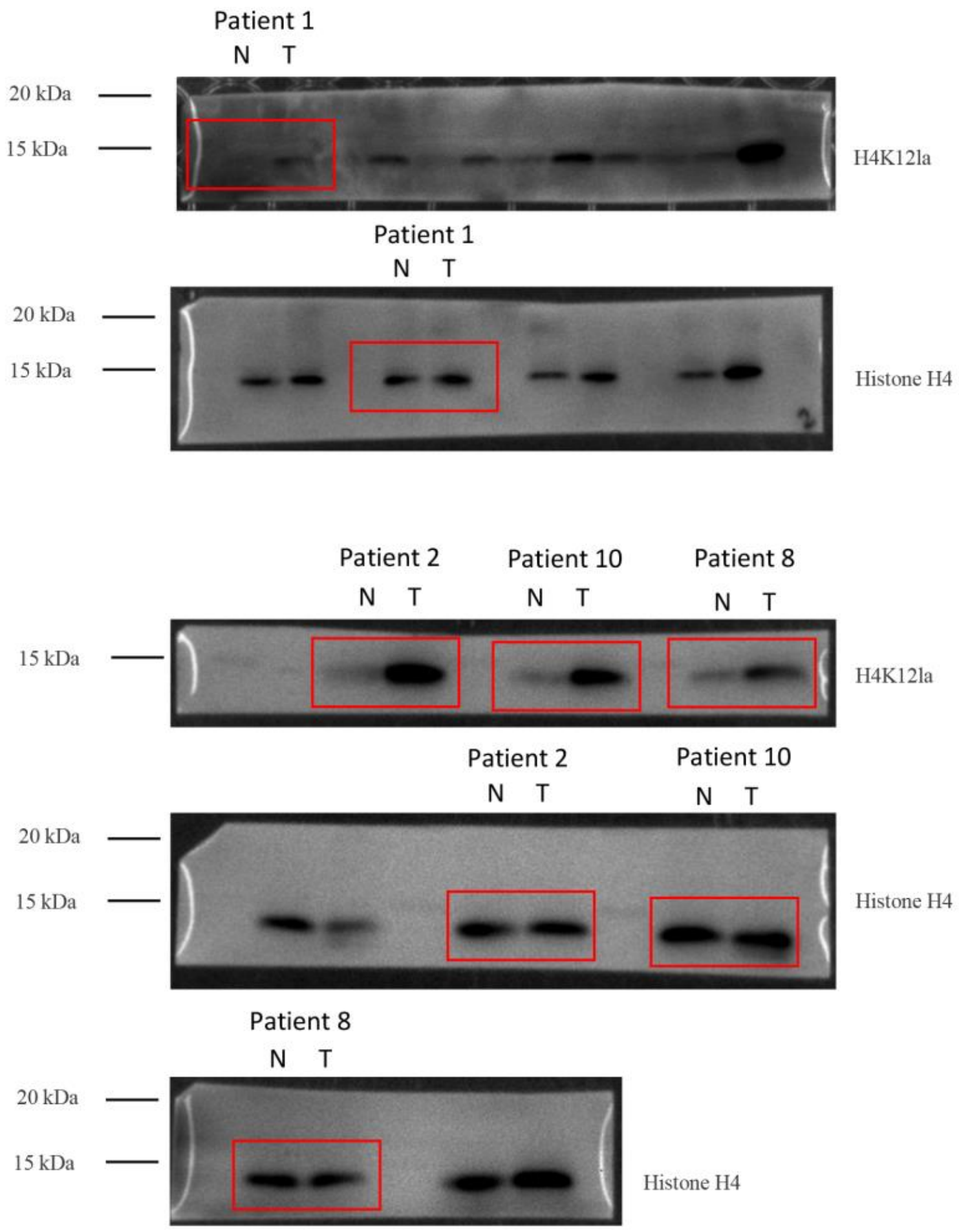

Uncropped gels for Western Blots in Figure 2

Figure 2A

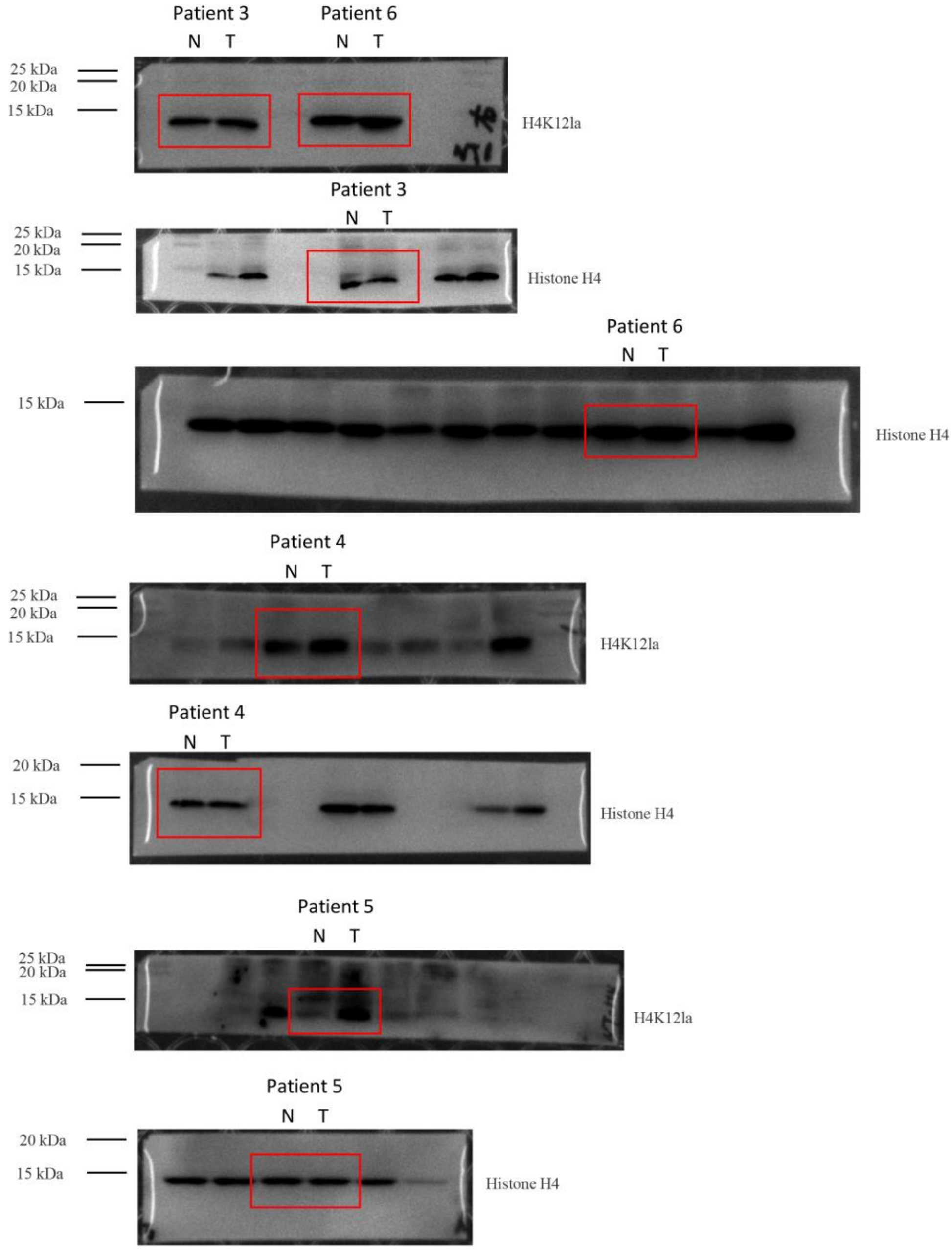

Uncropped gels for Western Blots in Figure 2

Figure 2A

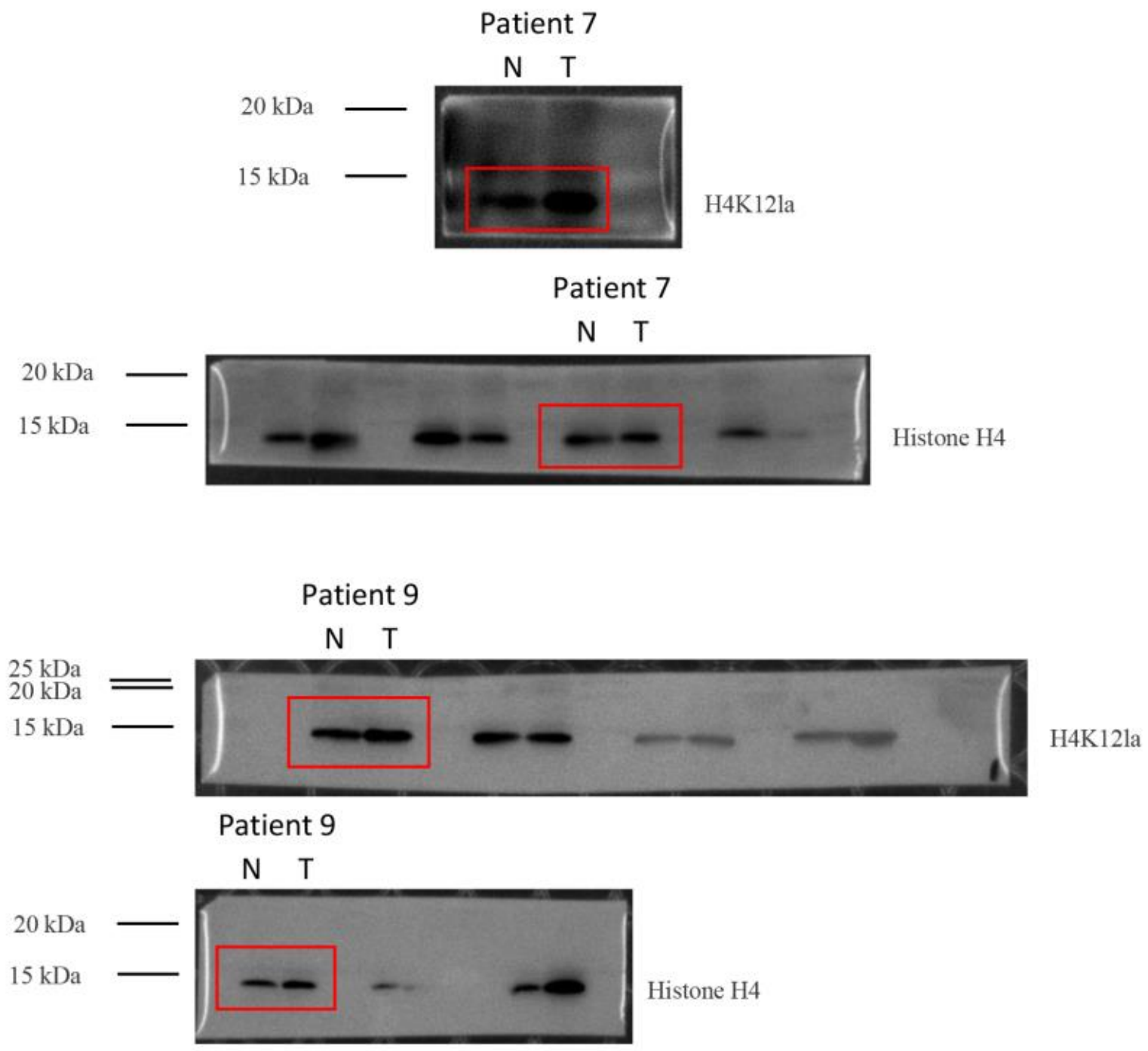

Uncropped gels for Western Blots in Figure 3

Figure 3A

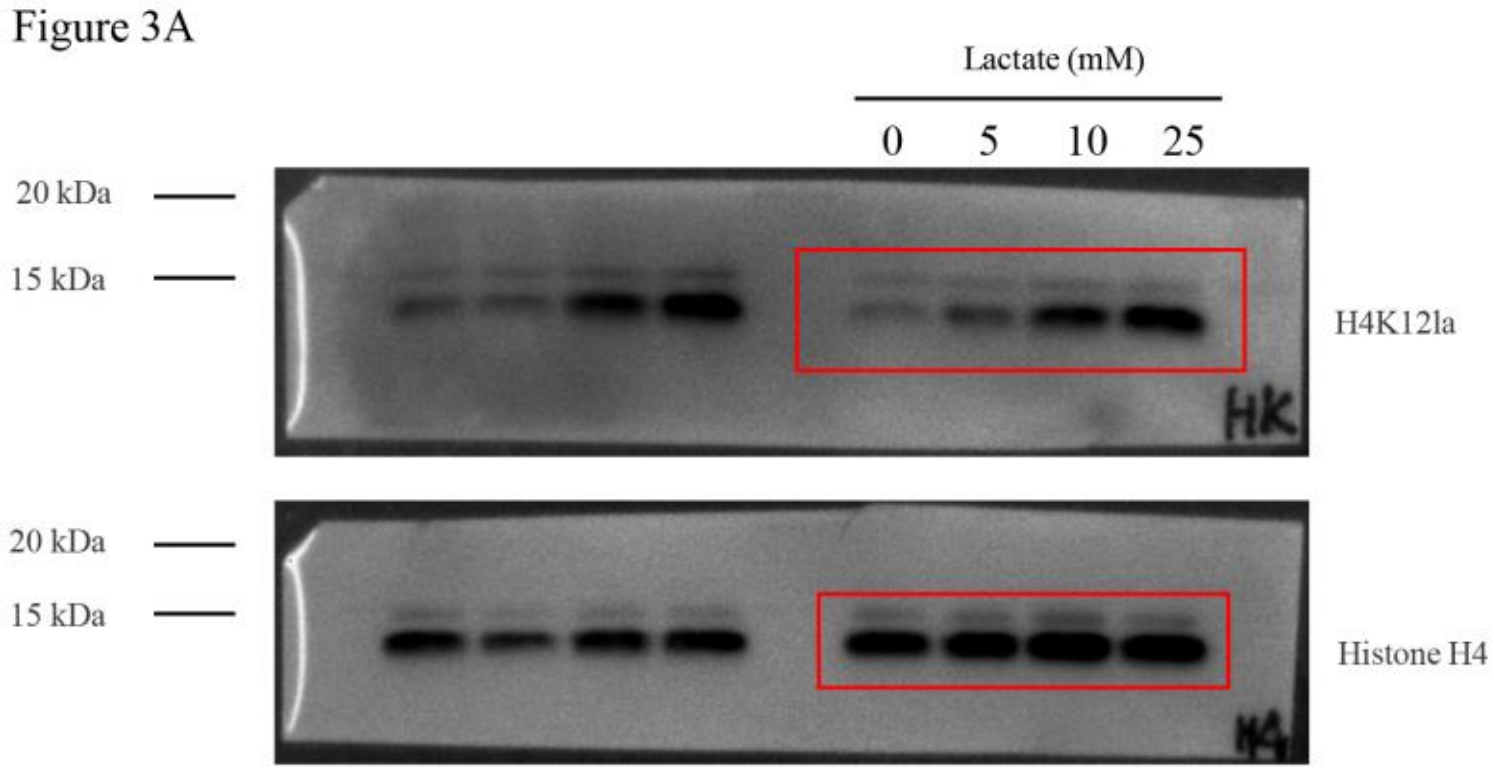

Figure 3D

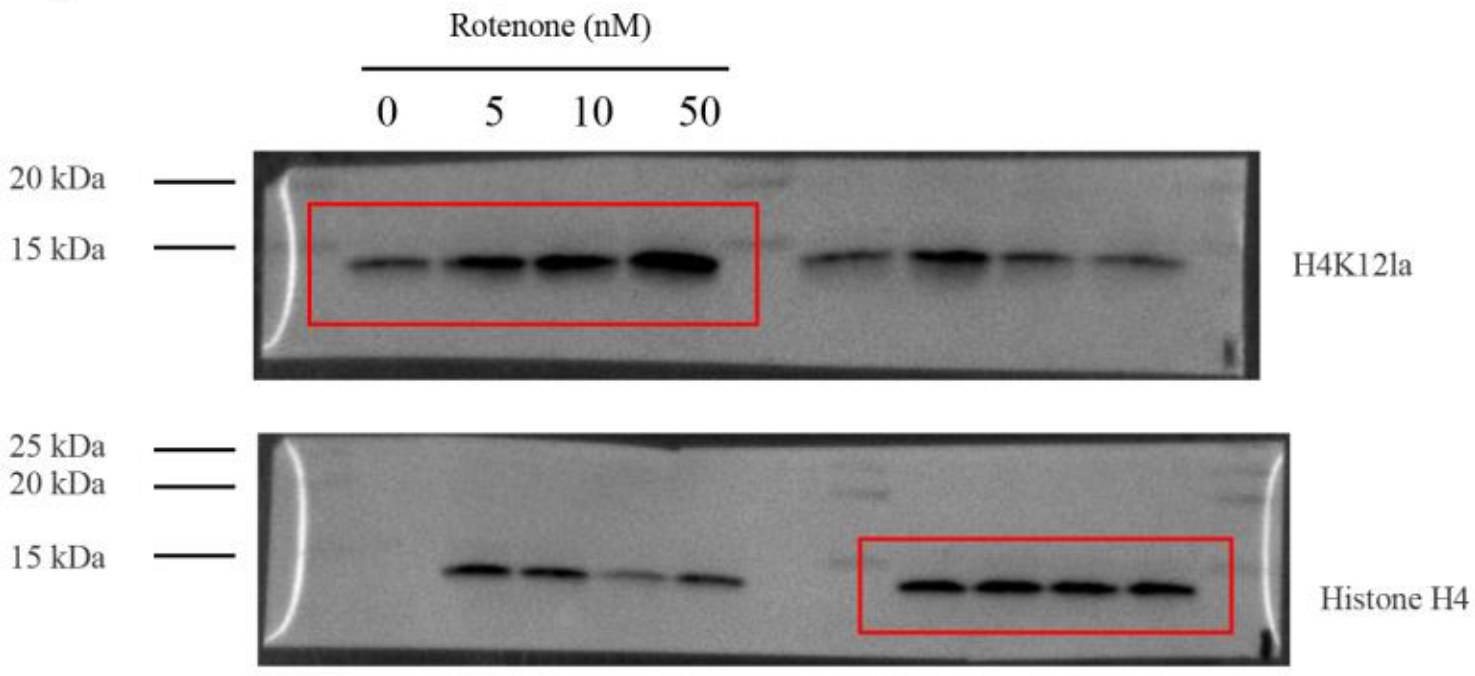

Figure 3F

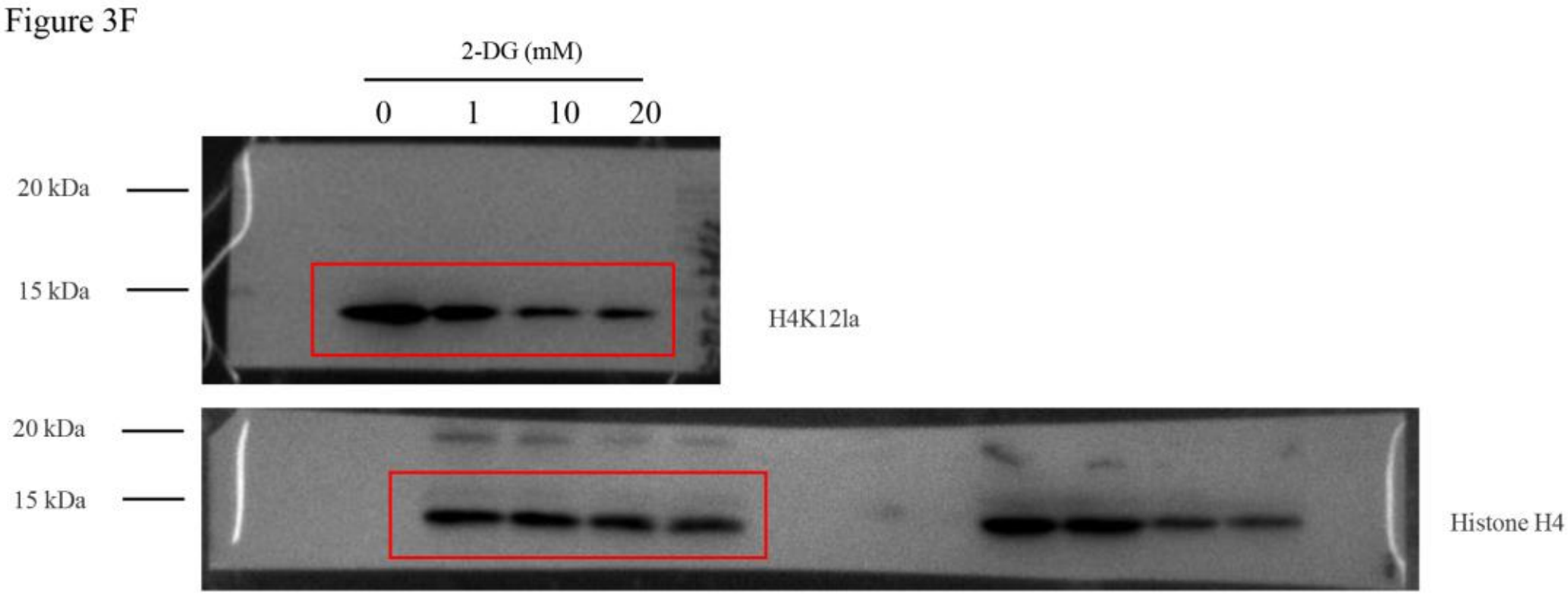

Uncropped gels for Western Blots in Figure 3

Figure 3G

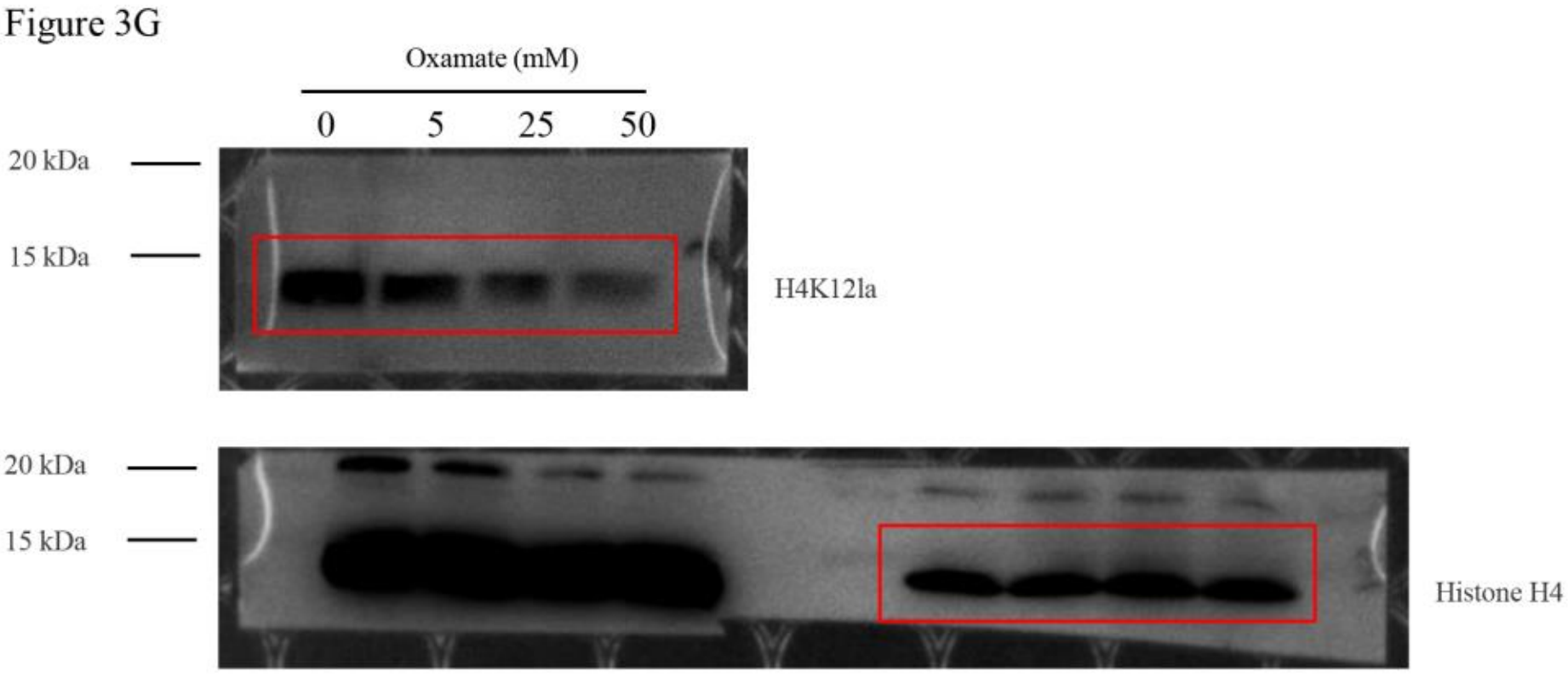

Figure 3H

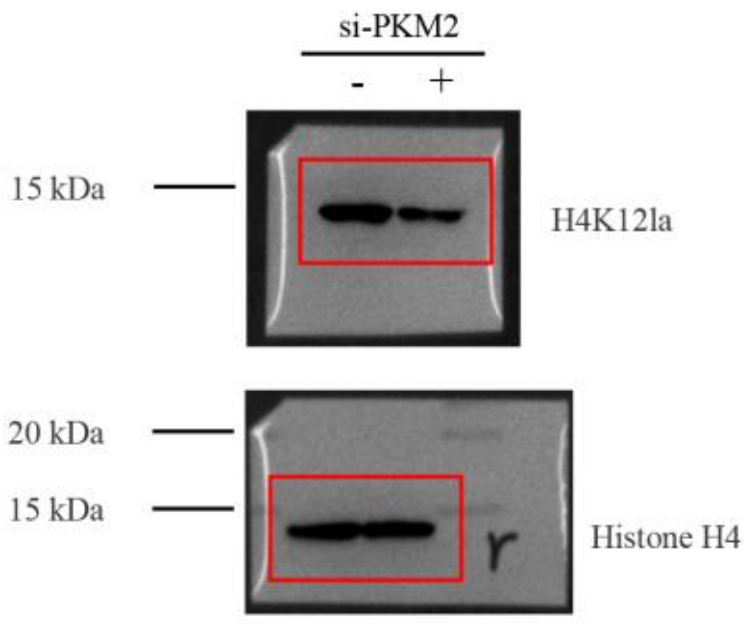

Figure 3I

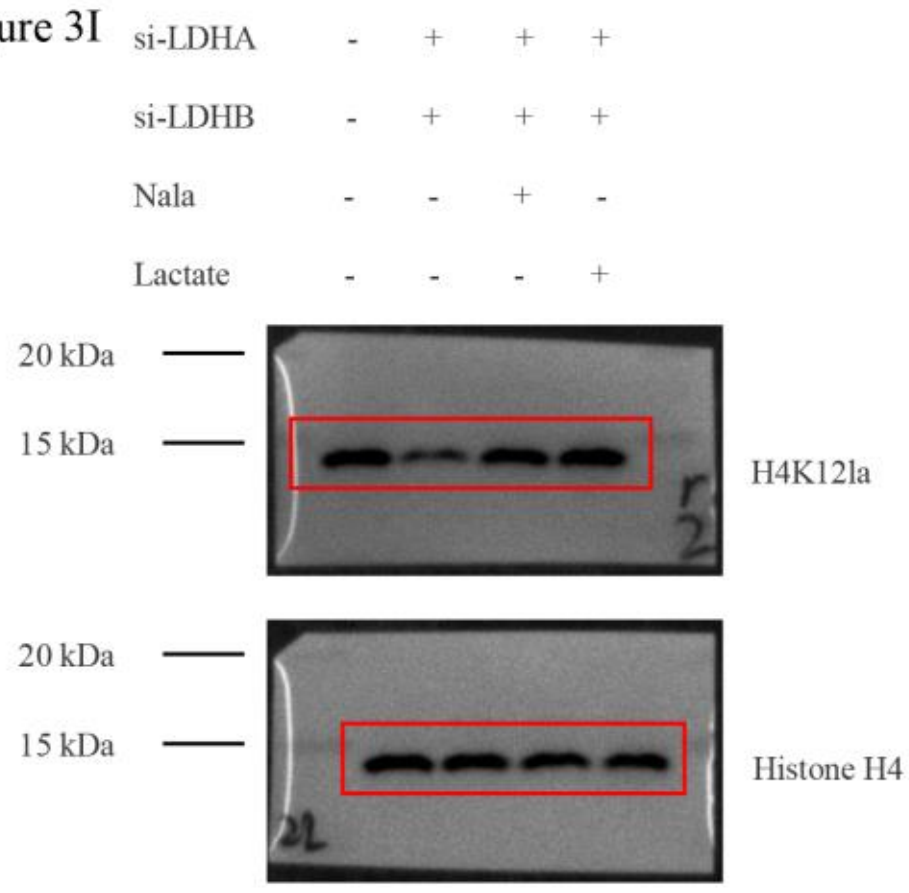

Figure 3J

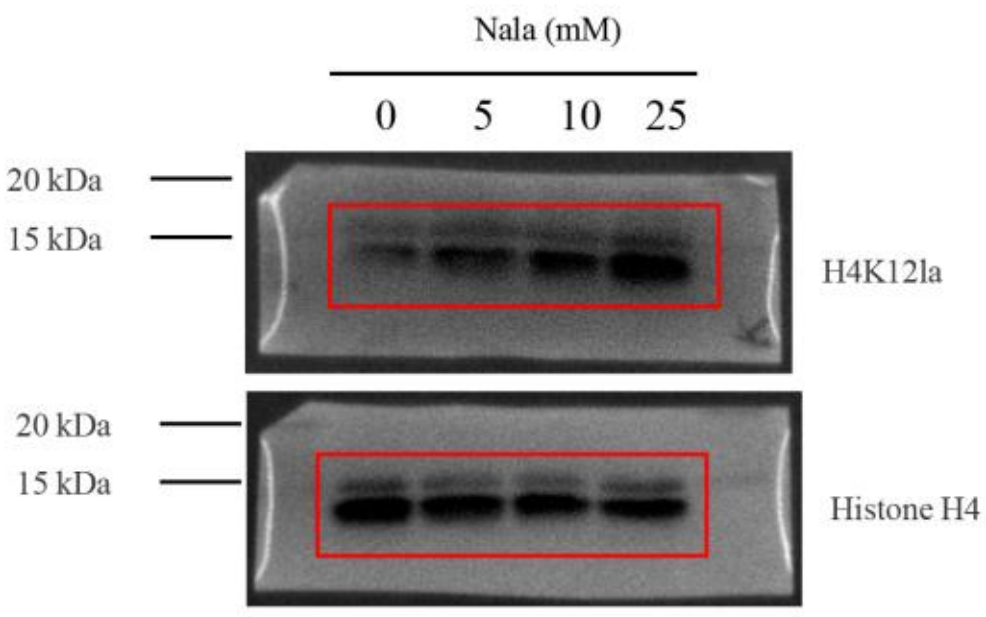

Uncropped gels for Western Blots in Figure 4

Figure 4A

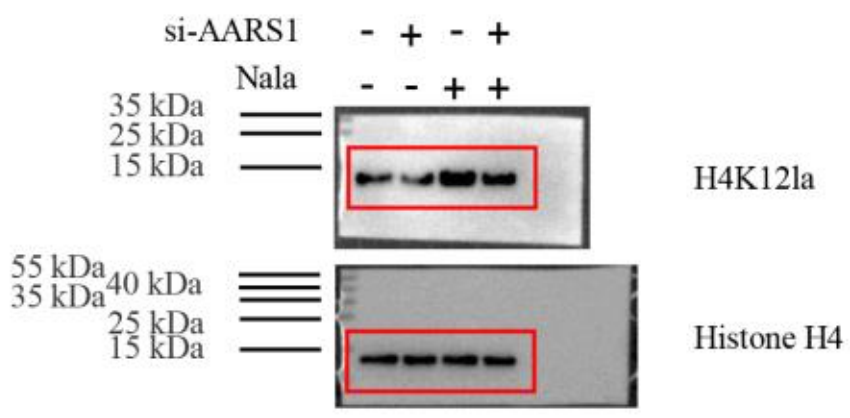

Figure 4B

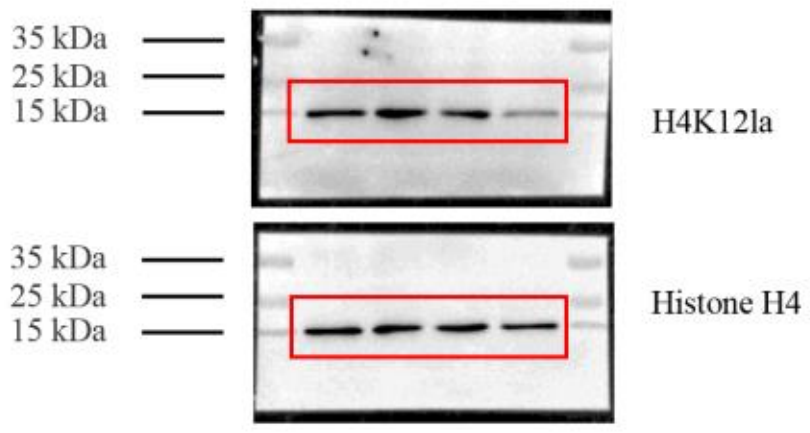

Figure 4C

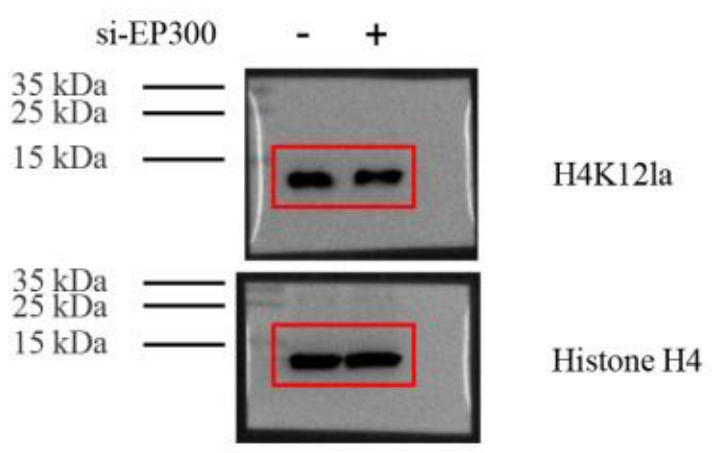

Figure 4D

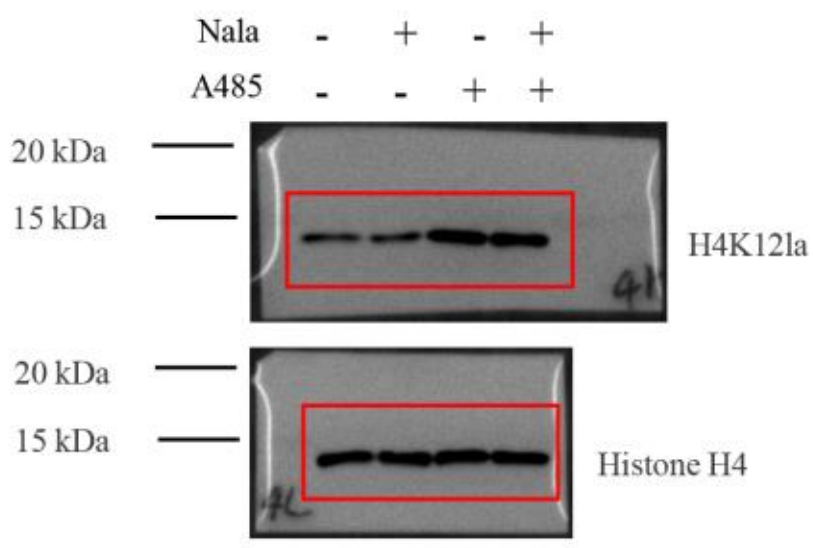

Figure 4E

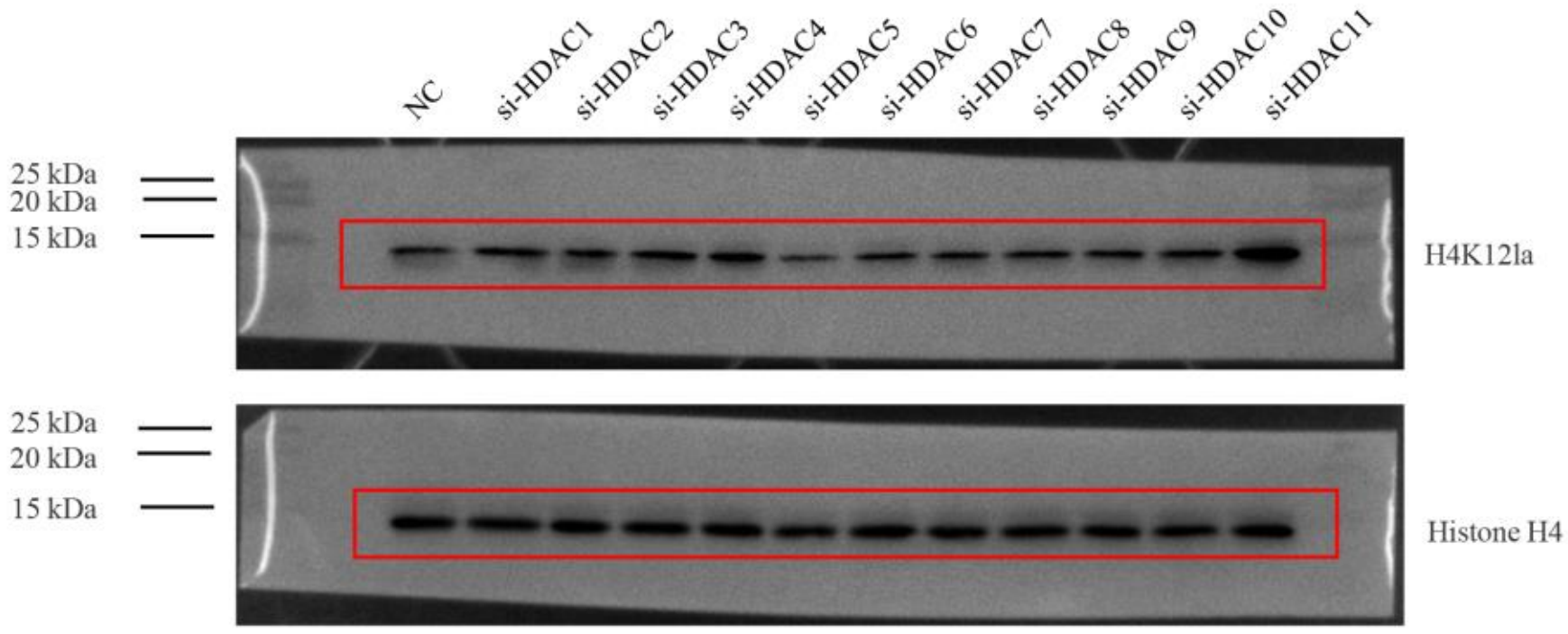

Uncropped gels for Western Blots in Figure 4

Figure 4F

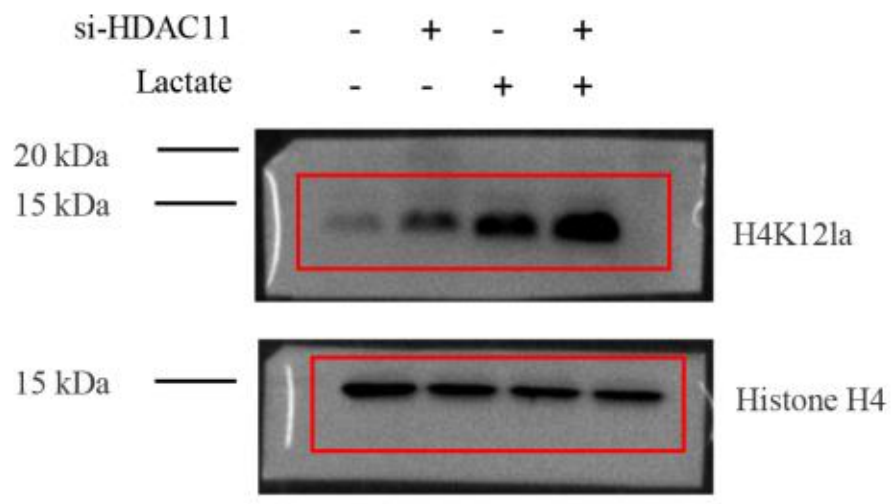

Figure 4G

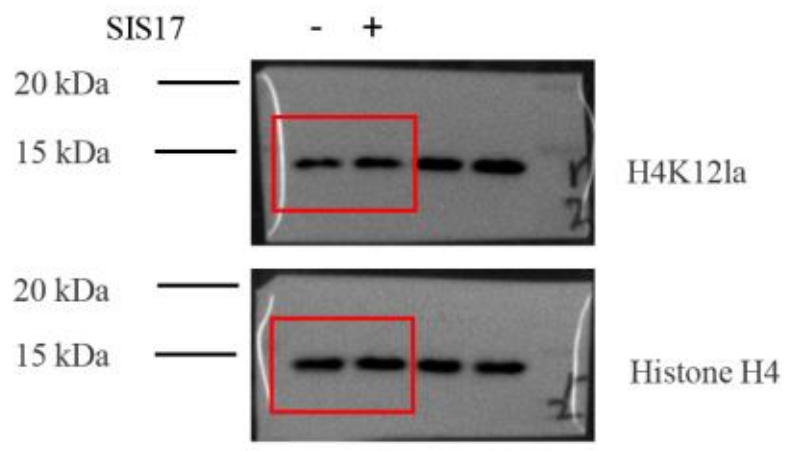

Uncropped gels for Western Blots in Figure 5

Figure 5H

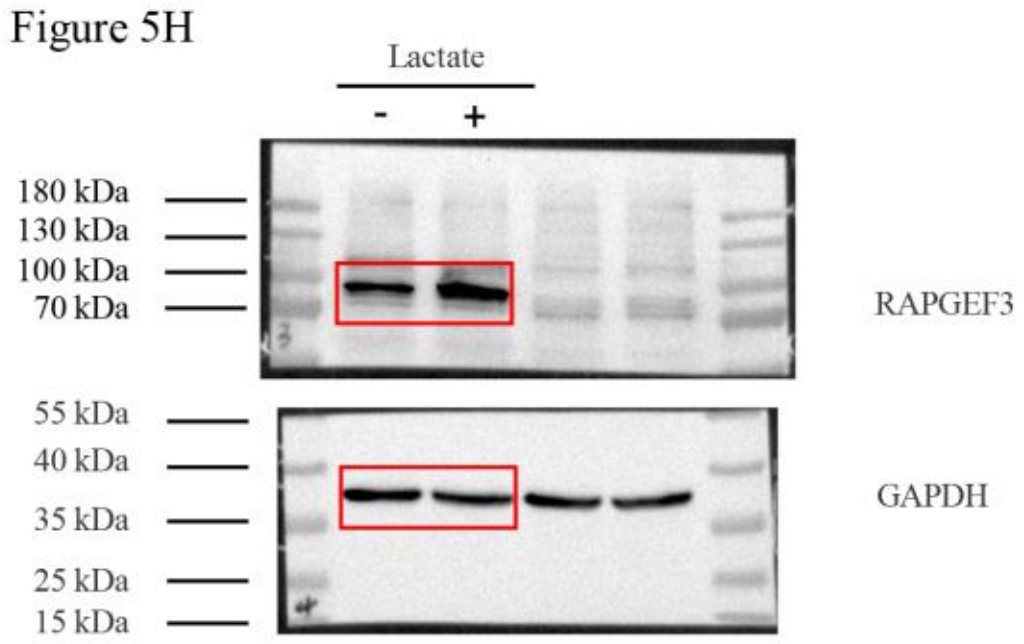

Figure 5I

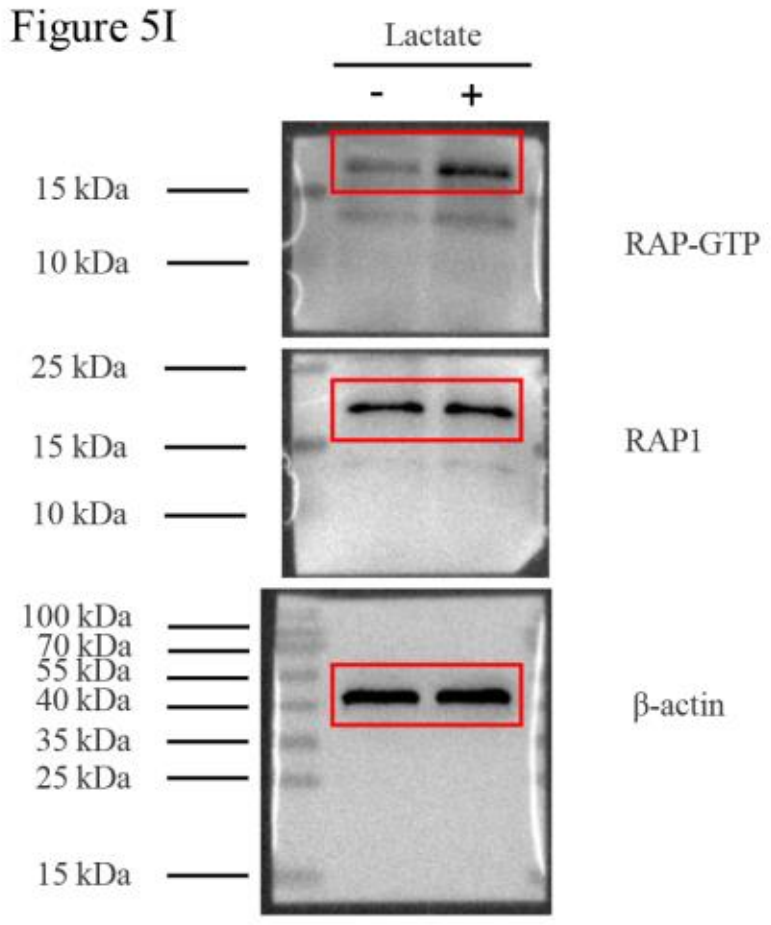

Uncropped gels for Western Blots in Figure 6

Figure 6A

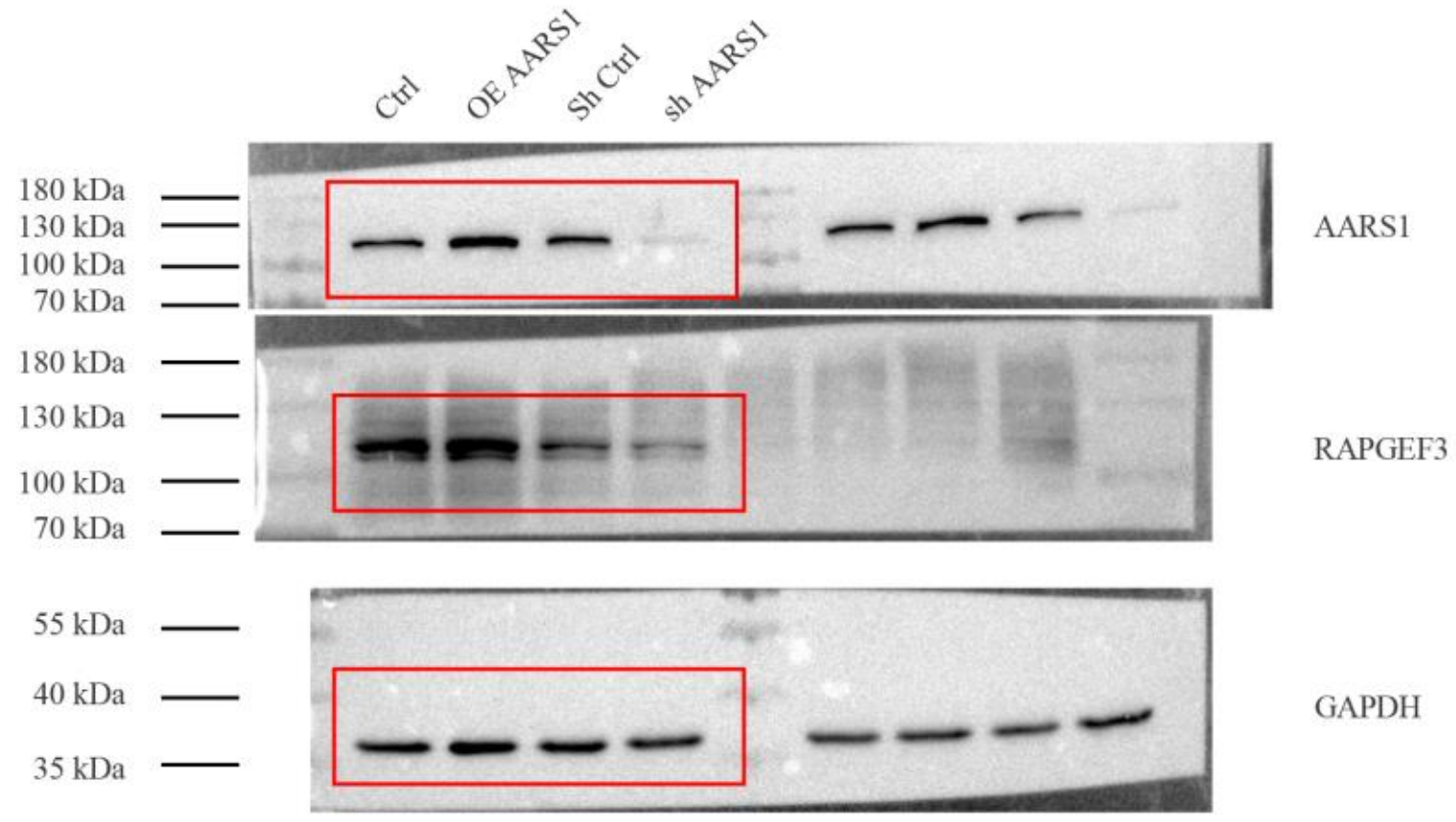

Uncropped gels for Western Blots in Figure S1

Figure S1C

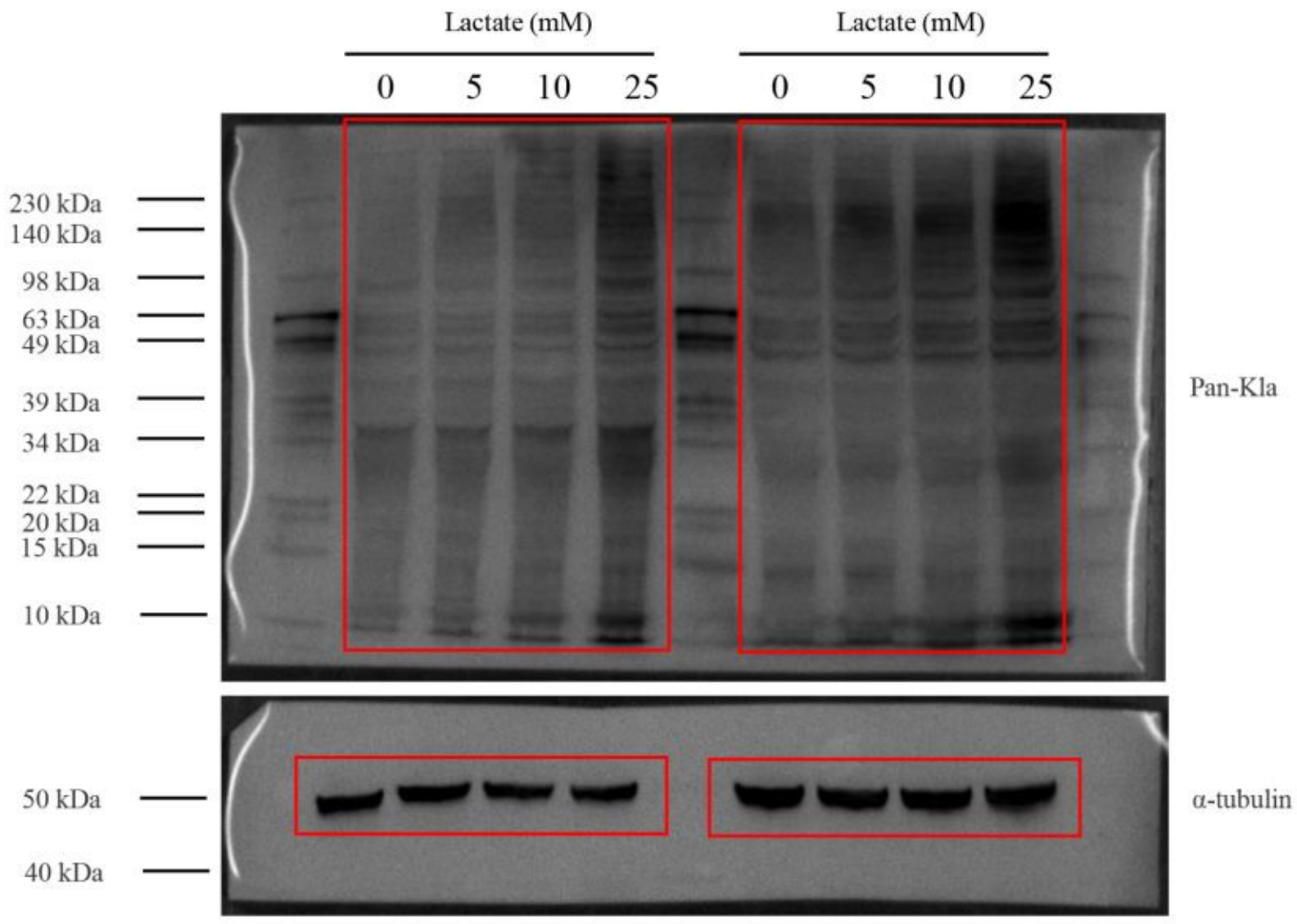

Figure S1D

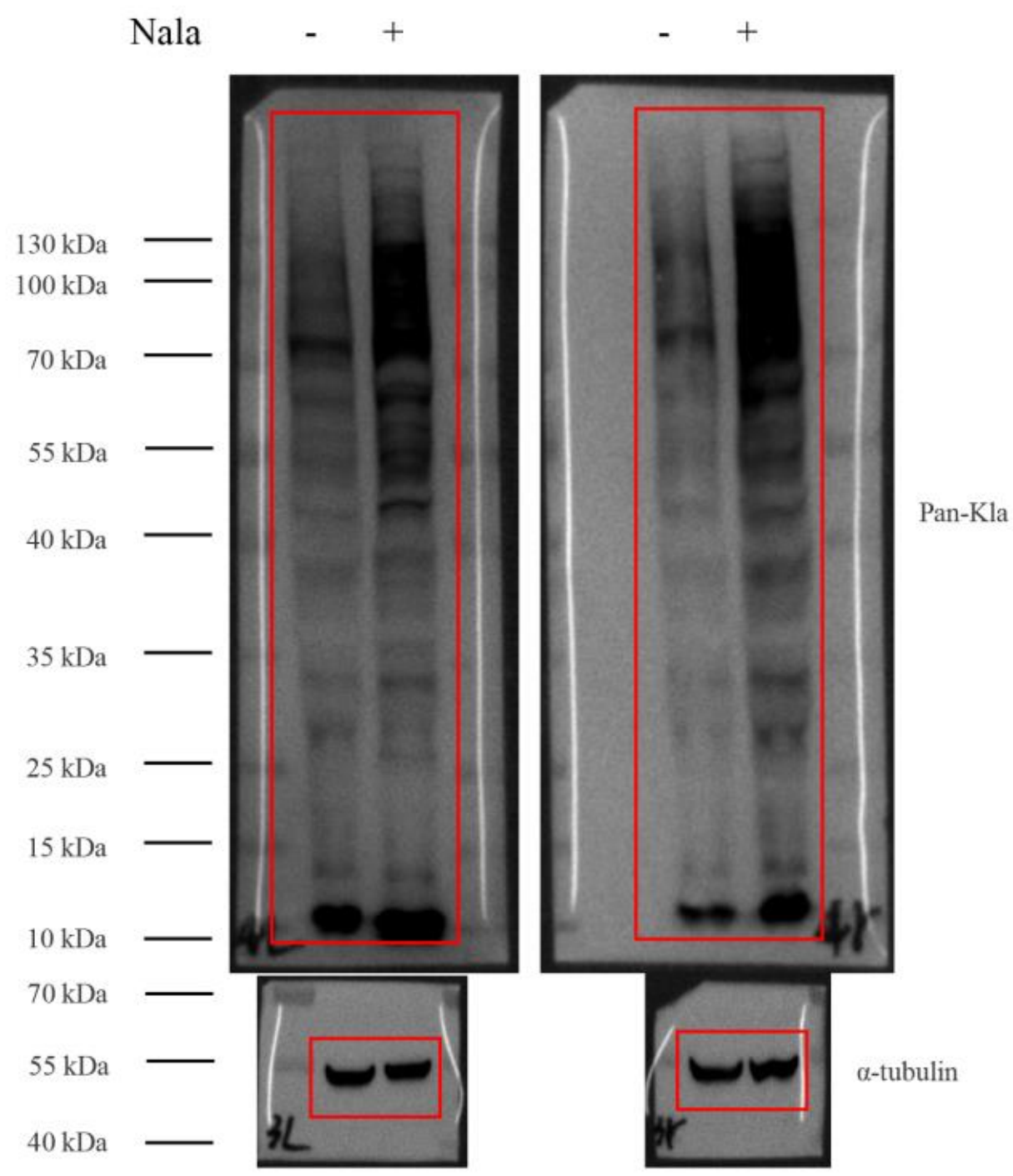

Uncropped gels for Western Blots in Figure S2

Figure S2A

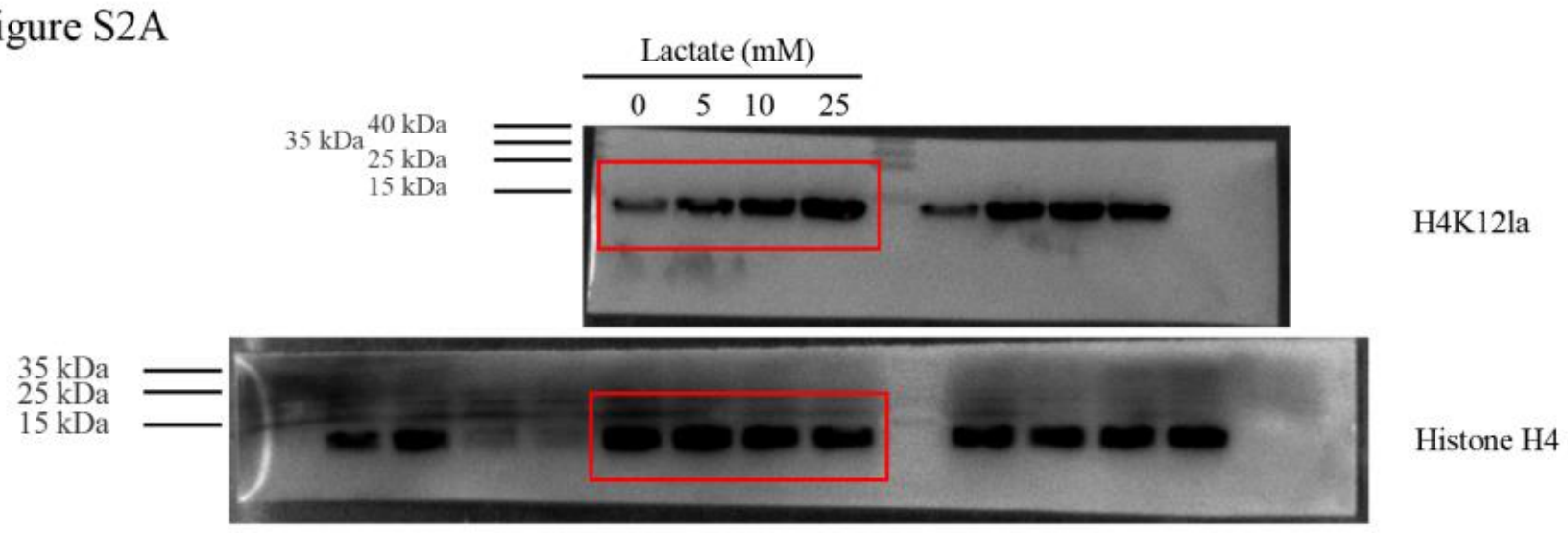

Figure S2B

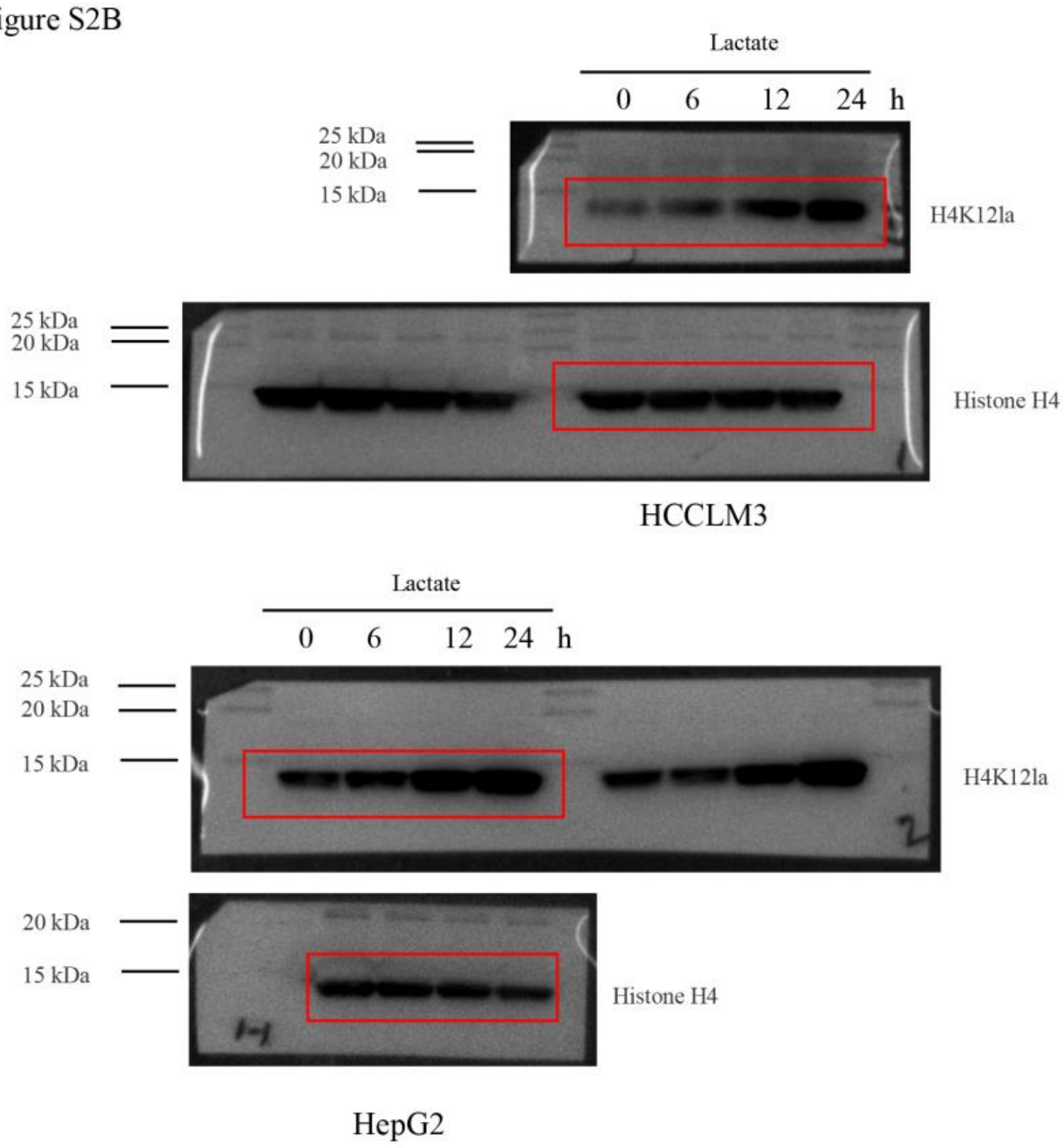

Uncropped gels for Western Blots in Figure S2

Figure S2D

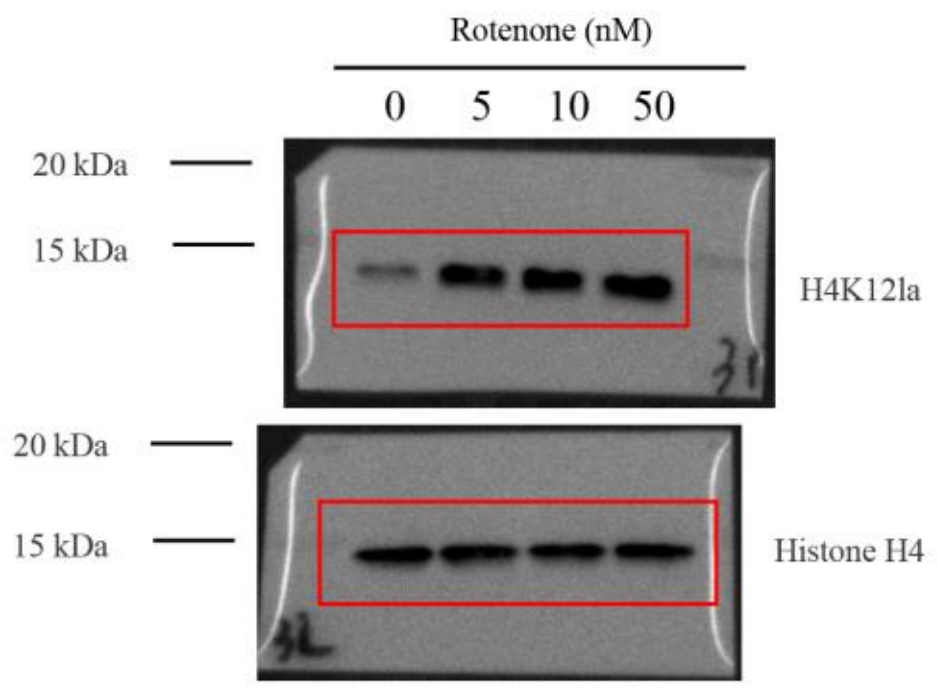

Figure S2F

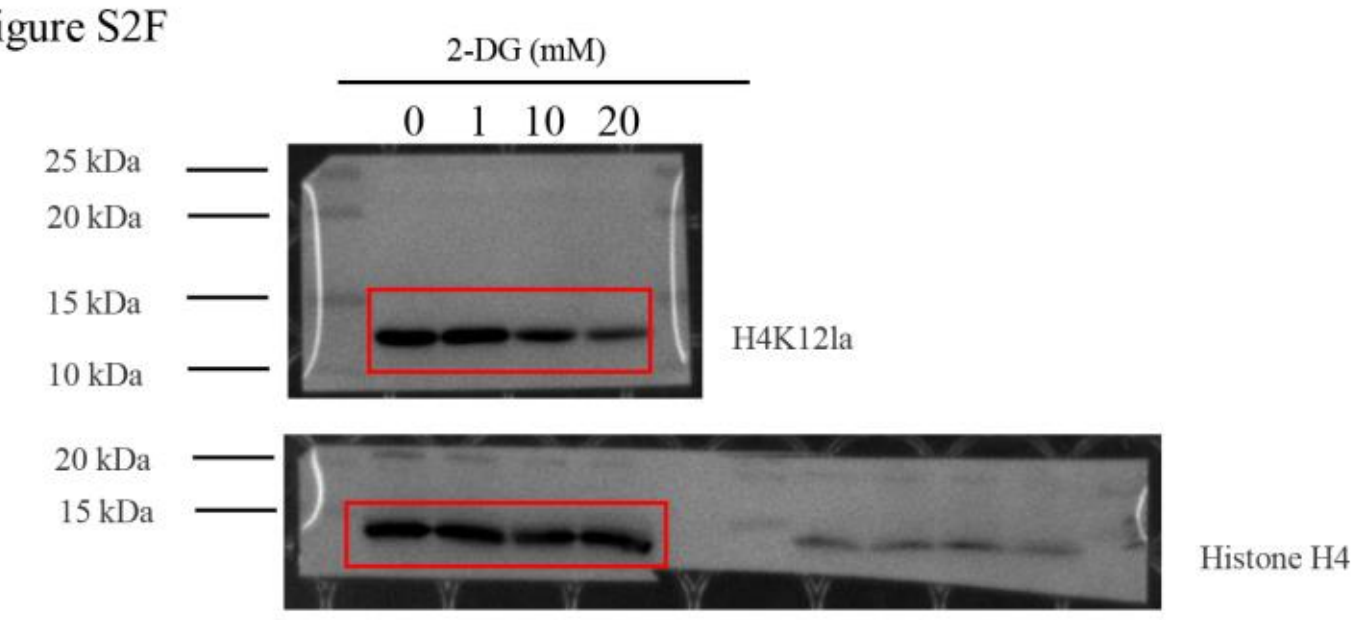

Figure S2G

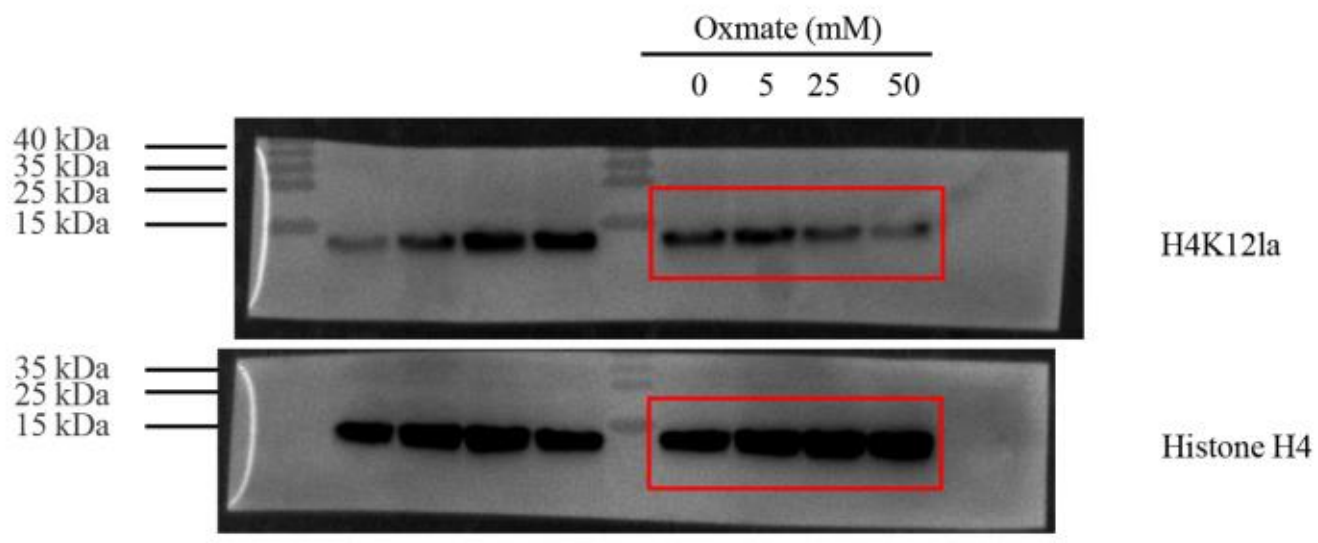

Uncropped gels for Western Blots in Figure S2

Figure S2I

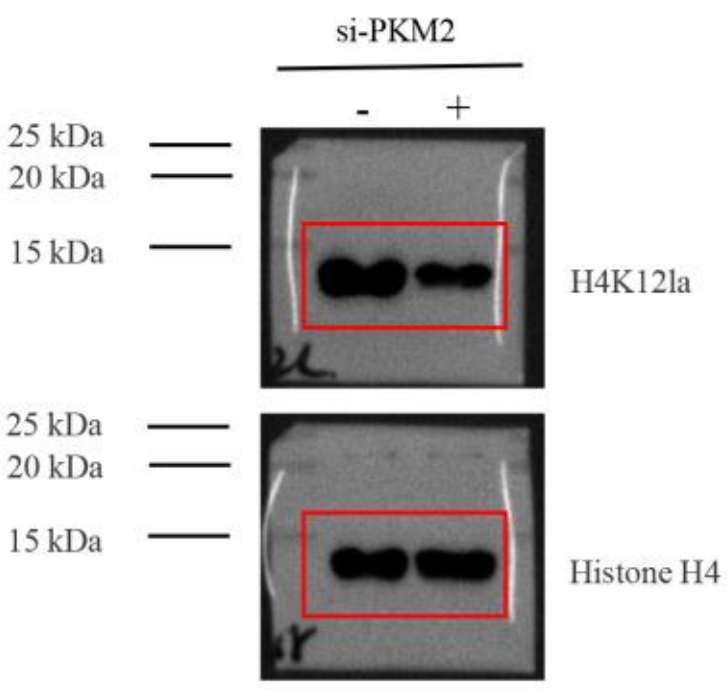

Figure S2L

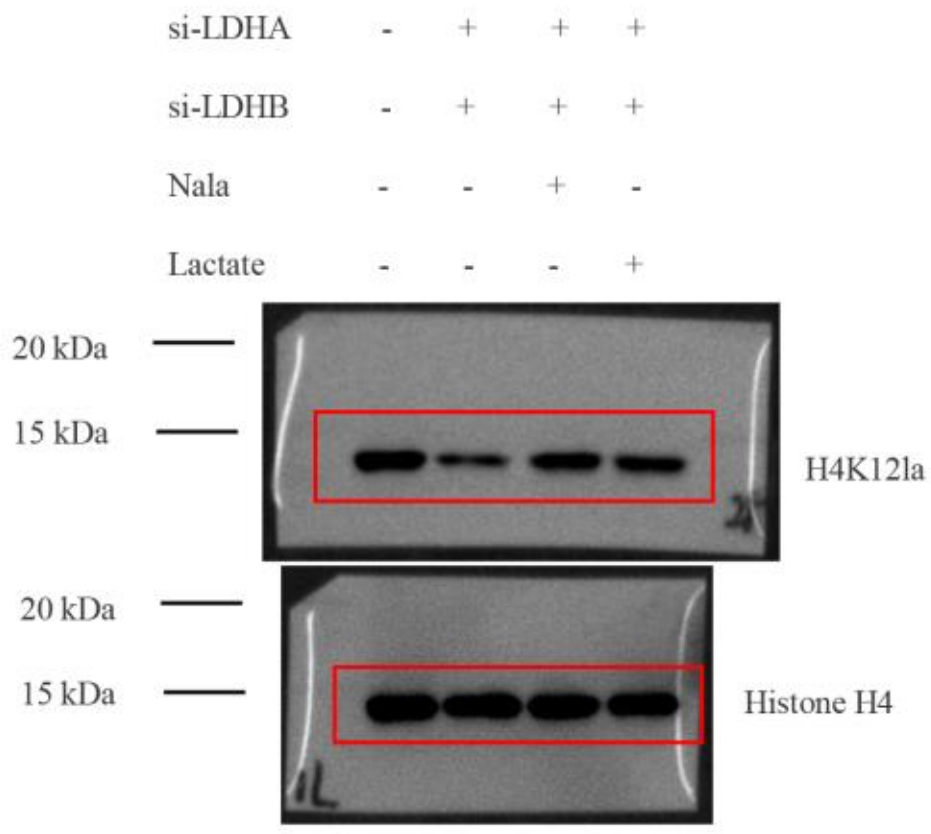

Figure S2M

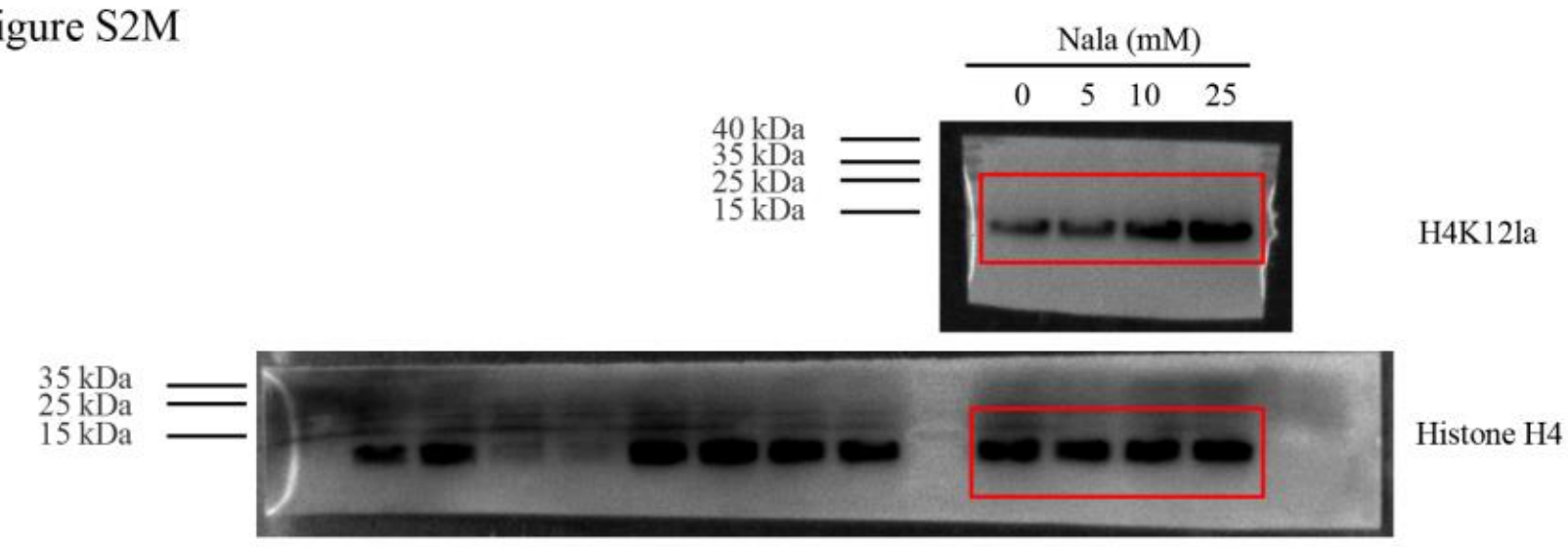

Uncropped gels for Western Blots in Figure S3

Figure S3A

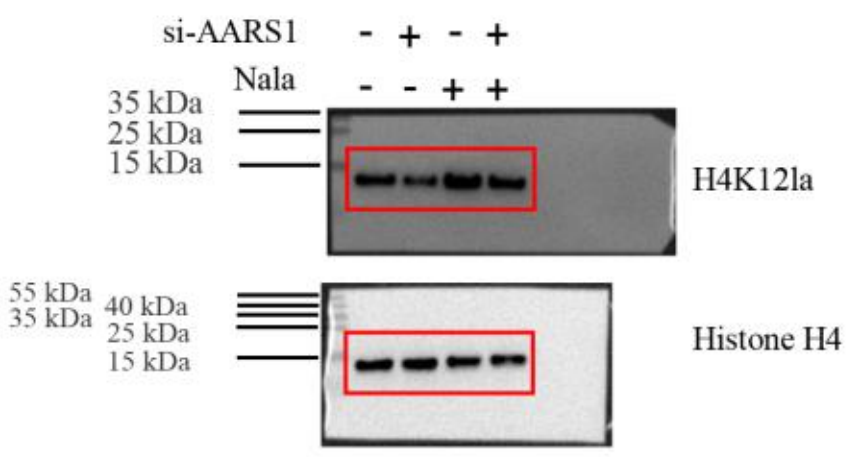

Figure S3B

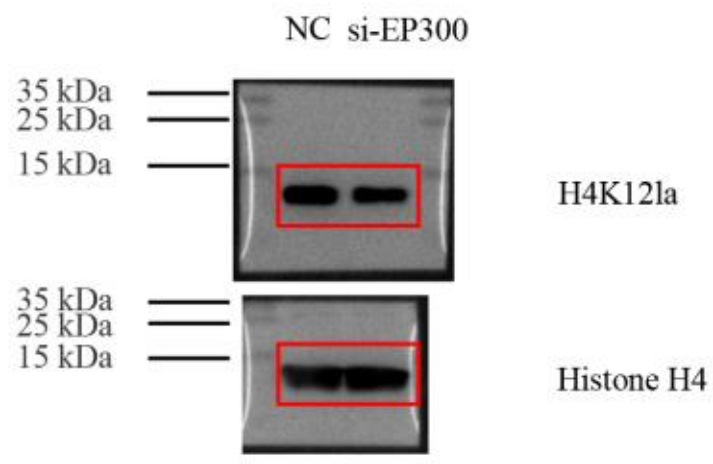

Figure S3D

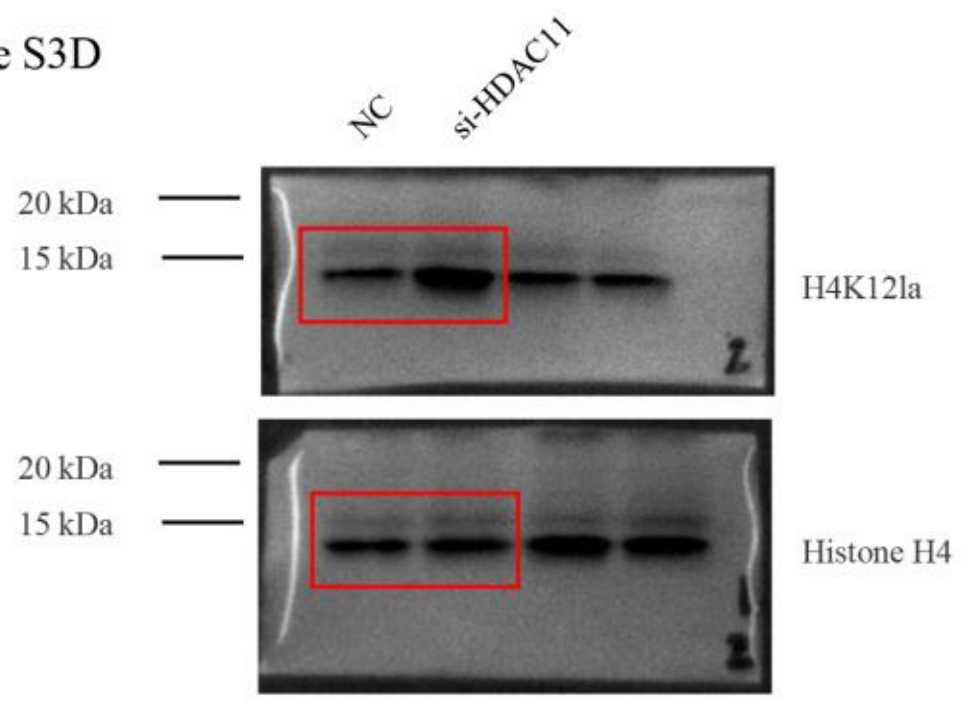

Supplement: Supplementary file 2 [file 41419_2026_9011_MOESM2_ESM.pdf]
